# TDP-43 regulates cholesterol biosynthesis by inhibiting sterol regulatory element-binding protein 2

**DOI:** 10.1038/s41598-022-12133-4

**Published:** 2022-05-14

**Authors:** Naohiro Egawa, Yuishin Izumi, Hidefumi Suzuki, Itaru Tsuge, Koji Fujita, Hitoshi Shimano, Keiichi Izumikawa, Nobuhiro Takahashi, Kayoko Tsukita, Takako Enami, Masahiro Nakamura, Akira Watanabe, Motoko Naitoh, Shigehiko Suzuki, Tsuneyoshi Seki, Kazuhiro Kobayashi, Tatsushi Toda, Ryuji Kaji, Ryosuke Takahashi, Haruhisa Inoue

**Affiliations:** 1 Center for iPS Cell Research and Application (CiRA), Kyoto University, Kyoto, Japan; 2 Department of Neurology, Graduate School of Medicine, Kyoto University, Kyoto, Japan; 3 iPSC-Based Drug Discovery and Development Team, RIKEN BioResource Research Center (BRC), Kyoto, Japan; 4 Department of Clinical Neuroscience, The University of Tokushima Graduate School, Tokushima, Japan; 5 Department of Plastic and Reconstructive Surgery, Graduate School of Medicine, Kyoto University, Kyoto, Japan; 6 Department of Endocrinology and Metabolism, Faculty of Medicine, University of Tsukuba, Ibaraki, Japan; 7 Department of Applied Biological Science, Graduate School of Agriculture, Tokyo University of Agriculture and Technology, Tokyo, Japan; 8 Medical-Risk Avoidance Based On iPS Cells Team, RIKEN Center for Advanced Intelligence Project (AIP), Kyoto, Japan; 9 Medical Innovation Center, Graduate School of Medicine, Kyoto University, Kyoto, Japan; 10 Division of Neurology/Molecular Brain Science, Kobe University Graduate School of Medicine, Kobe, Japan; 11 Department of Neurology, Graduate School of Medicine, The University of Tokyo, Tokyo, Japan

**Keywords:** Neurology, Motor neuron disease, Neurodegeneration

## Abstract

Dyslipidemia is considered an essential component of the pathological process of amyotrophic lateral sclerosis (ALS), a fatal motor neuron disease. Although TAR DNA Binding Protein 43 kDa (TDP-43) links both familial and sporadic forms of ALS and cytoplasmic aggregates are a hallmark of most cases of ALS, the molecular mechanism and the in vivo relation of ALS dyslipidemia with TDP-43 have been unclear. To analyze the dyslipidemia-related gene expression by TDP-43, we performed expression microarray and RNA deep sequencing (RNA-Seq) using cell lines expressing high levels of TDP-43 and identified 434 significantly altered genes including sterol regulatory element-binding protein 2 (SREBP2), a master regulator of cholesterol homeostasis and its downstream genes. Elevated TDP-43 impaired SREBP2 transcriptional activity, leading to inhibition of cholesterol biosynthesis. The amount of cholesterol was significantly decreased in the spinal cords of TDP-43-overexpressed ALS model mice and in the cerebrospinal fluids of ALS patients. These results suggested that TDP-43 could play an essential role in cholesterol biosynthesis in relation to ALS dyslipidemia.

## Introduction

Amyotrophic lateral sclerosis (ALS) is a fatal neurodegenerative disease characterized by selective motor neuronal (MN) death with cytosolic aggregates^1^ mainly consisting of TAR-DNA Binding Protein of 43 kDa (TDP-43)^2,3^. Clinical studies have shown that dyslipidemia and presymptomatic body fat are associated with the risk and prognosis of ALS patients^4–7^. High serum levels of cholesterol, or hyperlipidemia, have been reported to be protective against the prognosis of ALS^8–11^, and the presence of hypolipidemia precedes disease progression in ALS model mice^12,13^. In addition, glycolysis upregulation was reported to be protective against ALS progression in a *Drosophila* model of TDP-43 proteinopathy^14^. Furthermore, the onset of ALS in patients with antecedent hyperlipidemia is delayed by 6 months^15^, and dyslipidemia is presumed to be involved in other neurodegenerative diseases such as Huntington’s disease^16^. Genome-wide study of DNA methylation showed an altered cholesterol pathway in ALS patients^17^.

We previously reported that TDP-43 was elevated in both RNA and protein levels and that the expression of genes involved in sterol biosynthesis is decreased, in ALS MNs derived from induced pluripotent stem cells (iPSCs) generated from familial ALS patients with mutant TDP-43^18^, suggesting that TDP-43 may play a role in dyslipidemia in ALS. However, the molecular mechanism and the in vivo relevance of dyslipidemia to TDP-43 were unclear. In the current study, we analyzed cholesterol metabolism-associated gene expression profiles regulated by overexpressed TDP-43 using a cellular model and the amount of cholesterol in a TDP-43 overexpressed ALS mouse model and in cerebrospinal fluid of ALS patients, and we found that TDP-43 inhibited the activity of a master regulator of cholesterol biosynthesis, SREBP2, and the in vivo cholesterol level.

## Methods

### Cell culture and plasmid

DAP (triple affinity-purification tag; a biotinylation sequence and FLAG tags, and N-terminal epitope tag; 6-histidine epitope tag) -TDP-43-inducible 293 T Rex cells were cultured in Dulbecco’s modified Eagle’s medium (DMEM, glucose, 4.5 g/l) supplemented with Tet System Approved FBS (Takara Bio USA, CA, USA) at 37 °C in a 5% CO_2_/95% air atmosphere^19^. The pSyn-SRE-Luc vector was kindly provided by Dr. Elena Cattaneo (Univ. of Milan), and pFLAG-N-SREBP2 (1–481) was generously donated by Dr. Yoshihiro Yoneda (Osaka Univ.). pcDNA3.1-Myc-SREBP2 (1–3423) and pcDNA3.1-TDP-43 (1–1253) were generated using the In-Fusion HD cloning Kit (Takara Bio USA).

### Construction of doxycycline-inducible cell lines

To establish a cell line stably expressing DAP-TDP-43 upon doxycycline application, we used an Flp-In T-Rex Expression System (Thermo Fisher Scientific). Flp-In T-Rex 293 cells (293 T Rex) were transfected with pOG44 (Flp-recombinase expression vector) (Thermo Fisher Scientific) and DAP-TDP-43 pcDNA5/FRT (FLP Recombination Target) /TO (Tet-On) and cultured for 48 h in culture medium containing 200 μg/ml of hygromycin B (Thermo Fisher Scientific)^19^.

### Gene expression analysis

One hundred ng of total RNA extracted from DAP-TDP-43 cells was processed by using the Ambion WT Expression Kit (Thermo Fisher Scientific) and WT Terminal Labeling and Controls Kit (Thermo Fisher Scientific) according to the manufacturer’s instructions. The sample was then hybridized onto GeneChip Human Transcriptome Array 2.0 (Thermo Fisher Scientific). After washing and staining, the microarray was scanned by GeneChip Scanner 3000 7G (Thermo Fisher Scientific). The sample was re-hybridized onto a GeneChip Human Gene 1.0 ST Array (Thermo Fisher Scientific), and the microarray was washed, stained and scanned. Signal data were analyzed with Transcriptome Analysis Console software (Thermo Fisher Scientific) for the Human Transcriptome Array and Partek Genomic Suite software (Partek Inc., St. Louis, MO, USA). IPA analysis (QIAGEN, Germantown, MD, USA) and gene set analysis for genes found with a fold-change greater than 1.2 were performed using Benjamini Hochberg FDR (p < 0.05). The data were examined using Genespring GX software (Agilent Technologies, La Jolla, CA, USA) for the gene set analysis.

### RNA sequence analysis (RNA-seq)

We extracted total RNA from DAP-TDP-43 cells using the RNeasy Plus Kit (QIAGEN, Hilden, Germany). After the depletion of ribosomal RNA by Ribo-Zero Gold (Illumina, San Diego, CA, USA), libraries were prepared using the Illumina TruSeq Stranded Total RNA Sample Prep Kit (Illumina). The libraries were sequenced in 100 cycle Single-Read mode of HiSeq2500 (Illumina). All sequence reads were extracted in FASTQ format using BCL2FASTQ Conversion Software 1.8.4 in the CASAVA 1.8.4 pipeline. The number of sequence reads is listed in Supplementary Information (SI): Table S1. The sequence reads were mapped to hg19 reference genes, downloaded on 10 December 2012, using Tophat v2.0.8b, and quantified by RPKMforGenes, downloaded on 19 October 2012. Gene Ontology analysis was performed with GOstats and GOdb v2.14.0 in R package 3.1.0. To estimate transcript variants, Partek Genomics Suite v6.6 with Gencode v19 reference annotation was used. Mapping was performed using TopHat (CCB at JHU, USA).

### Quantitative reverse transcription PCR (qRT-PCR)

QRT-PCR was performed using SYBR green and analyzed with StepOne software v2.1. Primers used for the measurement of SREBP2, TDP-43, HMGCS1, HMGCR, SQLE, LDLR, DHCR24 and LXR mRNA in amplified cDNA from cells are listed in SI: Table S2.

### Immunoblots

Cells were harvested and lysed in buffer (50 mM Tris–HCl buffer, pH 7.6, 10 mM ethylenediaminetetraacetic acid, protease inhibitor cocktail (Roche, Basel, Switzerland)) and phosphatase inhibitor (Roche) containing 1% SDS. After sonication, each 100-μg sample of protein was subjected to SDS-PAGE (5–15% polyacrylamide gels, BIO CRAFT, Tokyo, Japan), with 2-ME, and separated proteins were transferred to PVDF. The membranes were incubated with primary antibodies, then by appropriate secondary antibodies, and they were finally visualized using ECL plus or ECL chemiluminescence (GE Healthcare, Chicago, IL, USA). The following primary antibodies were used in this assay: TDP-43 (Proteintech, #10782-1-AP, 1:1,000), β-actin (Sigma-Aldrich, #A5441, 1:5,000), SREBP2 (Cayman Chemical, #10007663, 1:200), LDL-R (Abcam, #ab30532, 1:100), SCAP (Protein tech, #12266-1-AP, 1:200), Insig-1 (Novus Biologicals, #NB110-55244, 1:500), S1P (Sigma-Aldrich, #HPA040702, 1:1,000) and S2P (Cell Signaling Technology, #2157S, 1:1,000).

### Immunocytochemistry

Cells were fixed in 4% paraformaldehyde (pH 7.4) for 10 min at room temperature and rinsed with PBS. The cells were permeabilized in PBS containing 0.2% Triton X-100 for 10 min at room temperature, followed by rinsing with PBS. Nonspecific binding was blocked with Blocking One HIST (Nacalai, Kyoto, Japan) for 10 min at room temperature. Cells were incubated with primary antibodies overnight at 4 °C, and then labeled with appropriate fluorescent-tagged secondary antibodies. DAPI (Thermo Fisher Scientific) was used to label nuclei. Acquisition of fluorescence images and quantification were performed using In Cell Analyzer 6500 (GE Healthcare). The following primary antibodies were used in this assay: SREBP2 (Abcam, Emeryville, CA, 1:50).

### Equipment and settings

The image acquisition tool LAS 4000 (GE Healthcare) was used for immunoblots, and images were processed on imageJ (https://imagej.nih.gov/ij/) software.

### Reporter assay

HEK293T cells were transiently transfected with SRE-luciferase reporter construct (pSynSRE, 1.0 μg) and pRL-SV40 (Promega, Madison, WI, USA) (0.2 μg) along with pcDNA3.1-CMV-TDP-43 (0, 0.5, 1.0, 2.0 μg) or pcDNA3.1 (2.0, 1.0, 0.5, 0 μg) using Lipofectamine LTX (Thermo Fisher Scientific). After 72 h, the cells were lysed for luciferase assay. The lysates were measured in triplicate using a Dual Luciferase Reporter Assay System (Promega) on Envision Multilabel Reader (PerkinElmer, Waltham, MA, USA). Relative activity was defined as the ratio of firefly luciferase activity to Renilla luciferase activity to normalize for transfection efficiency.

### Mice

Prp-TDP43A315T mice (B6Cg-Tg (Prnp-TARDBP*A315T) 95Balo/J, 010700) were purchased from The Jackson Laboratory (Bar Harbor, ME, USA). As described previously^20^, their asymptomatic stage is between 1 and 2 months of age. The onset of gait disorder appeared at ∼ 3 months of age in transgenic mutant TDP-43 mice and increased little by little over the next few months. The animals became paralyzed at ∼ 5 months of age, corresponding to symptomatic stage (gender-independent average); they were unable to move their hindlimbs or right themselves when placed on their backs. For cholesterol measurements, at 3 months of age (pre-symptomatic stage) and 4–6 months of age (symptomatic stage) the mice were perfused with cold PBS and spinal lipids were extracted with chloroform/methanol (2:1) according to the Folch technique^21^ at Skylight Biotech (Akita, Japan). All surgical procedures were performed according to the rules established by the Ethics Committee of Kyoto University. All experiments were performed in accordance with ARRIVE guidelines and relevant regulations.

### Human spinal fluid samples

For disease diagnosis, spinal fluid samples were obtained by lumbar puncture; initial pressure, cell count, total protein level, and glucose level were measured. The remaining samples were frozen at − 80 °C until further analysis. Spinal fluids were collected from both ALS patients (*N* = 20) and disease-control patients (*N* = 20). The entity providing the material and following the patients is Tokushima University Hospital. All experiments were performed in accordance with relevant guidelines and regulations.

### Cholesterol measurement

For quantitative analysis of cholesterol, cells were homogenized in PBS and lipids were extracted by modified Bligh and Dyer extraction method^21,22^. Briefly, 100 μl of chloroform plus methanol (2:1) was added to 100 μl of PBS including homogenized cells after counting the cell number. After centrifugation, the chloroform layer was collected and dried with a centrifugal dryer. The dry matter was lysed in 100 μl of ethanol to measure free cholesterol using a Cholesterol Assay kit (Cayman Chemical Company, Ann Arbor, MI, USA). Fluorescence signals were read with an Envision Multilabel Reader (PerkinElmer). Total lipids including sterols in spinal fluids from patients were extracted by the same method, with the extracts being subjected to LC–MS/MS-based quantification (Agilent 6400). Briefly, 100 μl of chloroform plus methanol (2:1) was added to 10 μl of spinal fluids mixed with 90 μl of PBS. After centrifugation, the chloroform layer was collected and dried with a centrifugal dryer. The dry matter was then lysed in 100 μl of ethanol, filtered with a 0.2-μm centrifugal filter tube, and measured for cholesterol by the use of Agilent 6400.

### Statistics

All data are shown as mean ± s.e.m. or ± s.d. Two group-analyses were performed using unpaired two-tailed Student’s *t*-test or paired t-test. One- or two-way ANOVA was performed for each comparison, followed by Tukey’s post hoc tests for evaluation of pair-wise group differences. A *p* value < 0.05 was considered statistically significant. In a comparative analysis of the amount of cholesterol, an alternative hypothesis was applied, namely that sample data could differ between the two groups by 10% (α = 0.05, β = 0.2, power = 0.8 and effect size = 10% of the average), and the sample size was then determined. Analyses were performed by JMP (SAS Institute Inc., Cary, NC, USA) and Excel Tokei (Social Survey Research Information, Tokyo, Japan).

### Ethics approval and consent to participate

This study was approved by the Institutional Review Boards of Kyoto University and Tokushima University (Approval number: 2572-6, reference year: 2022), and all surgical procedures for animal studies were performed according to the rules set forth by the Ethics Committee of Kyoto University (Approval number: 17-91-11, reference year: 2022). Written informed consent was received from the participants prior to inclusion in the study. Samples from the participants were identified by numbers, not by names.

### Consent for publication

Written informed consent for publication was received from each participant.

## Results

### Gene expression and transcriptome profiling under high levels of TDP-43

**Figure 1:**
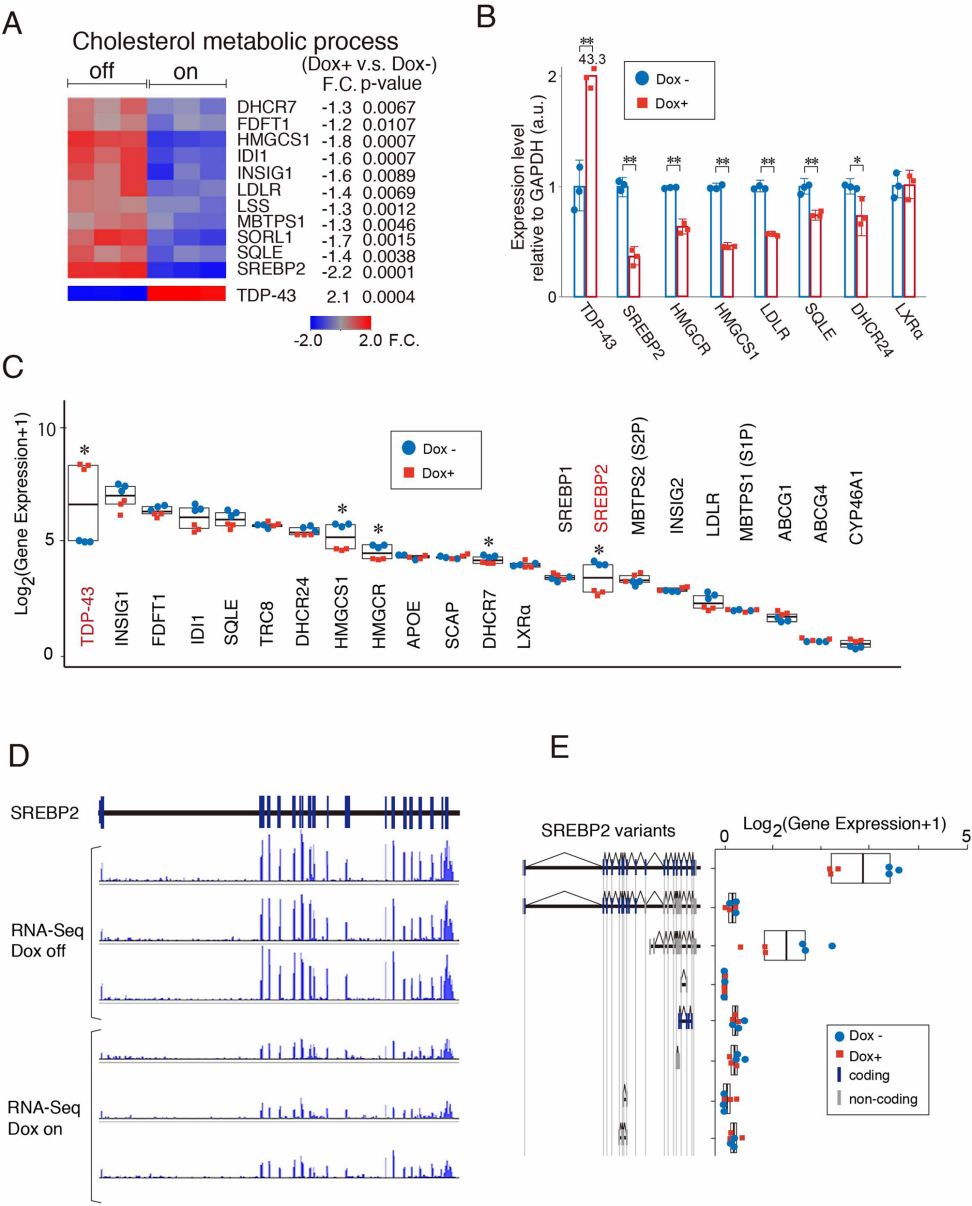
Gene expression and transcriptome profiling under TDP-43 induction. (**A**) A heat map showing decreased expression of genes related to the cholesterol metabolic process under TDP-43-overexpressed condition in the expression microarray in HEK293T Rex cells stably expressing doxycycline (Dox)-induced DAP-TDP-43 (Dox on (+, n = 3) vs. off (−, n = 3) condition, fold change (F.C.) < − 1.2). (**B**) QRT-PCR showed that mRNA levels of target genes of SREBP2 were decreased under TDP-43-overexpressed condition. *p < 0.05, **p < 0.01, t-test, error bars = s.d. For each independent experiment, n = 3. (**C**) Deep RNA sequencing analysis (n = 3 each) of genes related to cholesterol metabolism. Tukey, *p value < 0.05. (**D**) RNA-seq reads under Dox on or off condition. (**E**) Transcript variants of SREBP2 under Dox-on or -off condition.

To analayze dyslipidema-related gene expression by TDP-43, we performed expression microarray and RNA sequencing (RNAseq) using 293 T Rex cells stably expressing doxycycline-inductive DAP-TDP-43 (SI: Supplementary Fig. 1A)^19^. The microarray showed that elevated TDP-43 downregulated the expression of genes related to cholesterol biogenesis, including SREBP2, HMGCS1 and LDLR, with a significantly decreased cholesterol metabolic process as a gene ontology (GO) term (Fig. 1A and SI: Table S3). SREBP2 was a top upstream transcriptional regulator of the altered gene expressions caused by overexpressed TDP-43 (SI: Table S4). QRT-PCR showed that TDP-43 reduced the mRNA levels of genes related to cholesterol biogenesis including HMGCS1, HMGCR and LDLR as well as SREBP2 (Fig. 1B). We further performed RNAseq and identified 434 genes that were significantly altered under doxycycline-inductive TDP-43 (Tukey’s test, *p* < 0.05, SI: Table S5). Among them, elevated TDP-43 significantly decreased the expression levels of HMGCS1, HMGCR, DHCR7 and SREBP2 without biasing the splicing variants of SREBP2 (Fig. 1C–E). Taken together, TDP-43 could regulate the expression of genes related to cholesterol biosynthesis and their master regulator SREBP2.

### Overexpressed TDP-43 decreases cholesterol biogenesis by inhibiting SREBP2 activity

**Figure 2:**
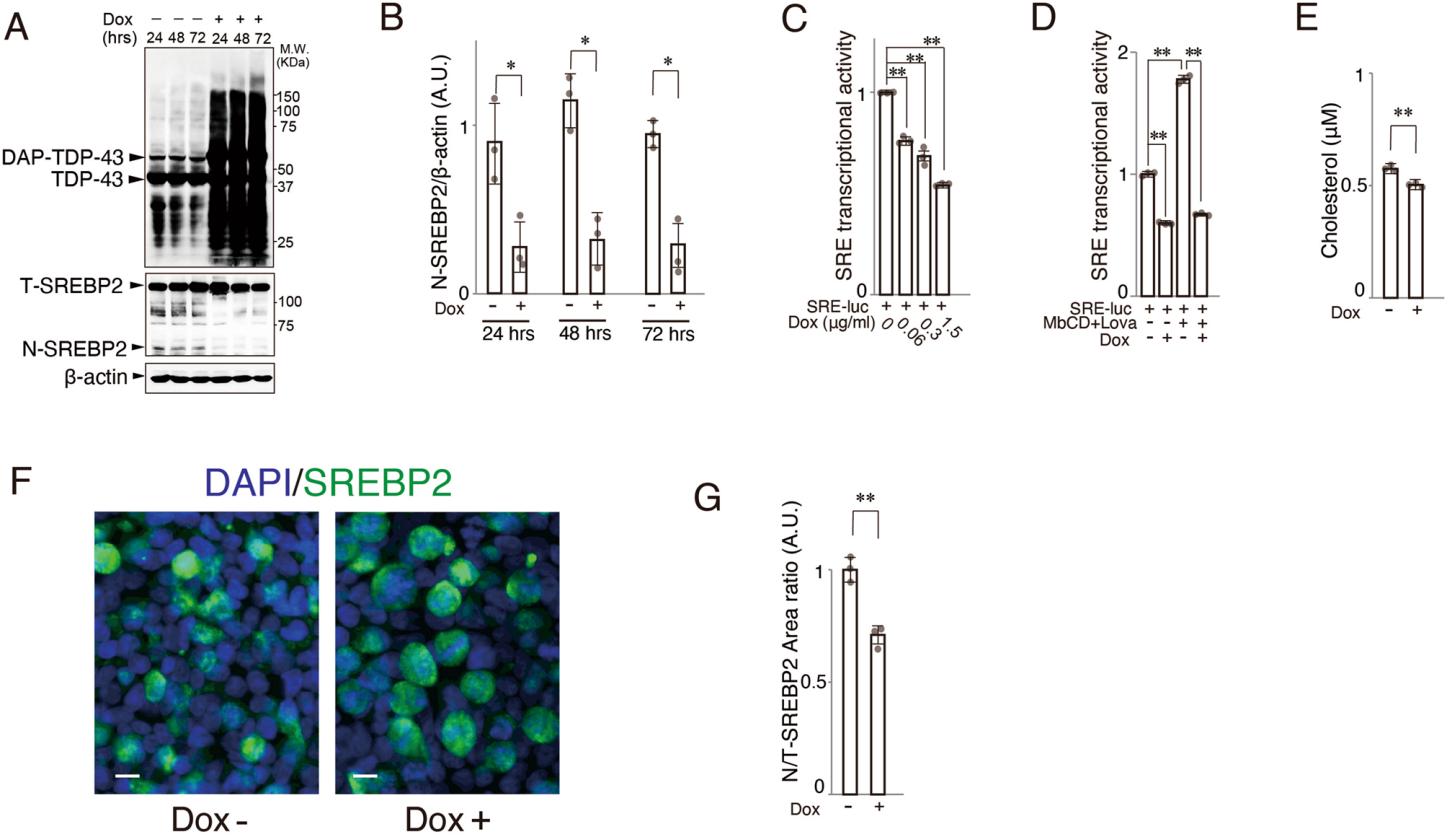
TDP-43 inhibits SREBP2 transcriptional activity via sterol-regulatory elements. (**A**) Immunoblots of lysates from 293 T Rex cells treated with Dox. The expression levels of N-SREBP2 were decreased at the indicated times after Dox (1.5 μM) treatment. Three panels were cropped from original blots presented by Supplementary Fig. 2. (**B**) Quantification of the cleaved N-terminal form of SREBP2 relative to β-actin; *p < 0.05, **p < 0.01, t-test, error bars = s.d. n = 3. (**C**) HEK293T Rex cells stably expressing doxycycline (Dox)-induced DAP-TDP-43 were transfected with pSyn-SRE-Luc and pRL-SV40 and treated with indicated concentrations of Dox and lysed for dual-luciferase assay to detect sterol-regulatory element (SRE)-activity; **p < 0.01, one-way ANOVA. (**D**) HEK293T Rex cells were transfected with pSyn-SRE-Luc and pRL-SV40 and treated with Dox and/or 10 mM methyl-beta-cyclodextrin (MbCD) and 0.4 μM Lovastatin (Lova); two-way ANOVA. (**E**) Amounts of free cholesterol in 293 T Rex cells (1 × 10^6^) 48 h after treatment with Dox; *p < 0.05, **p < 0.01, t-test, error bars = s.d. n = 3 for each assay performed. (**F**) Immunocytochemistry of SREBP2 after treatment with Dox (right panel) and without Dox (left panel). Green: SREPBP2, Blue: DAPI. Scale bar: 10 μm. (**G**) The ratio of the area of nuclear-localized SREBP2 (N) to that of total cell-localized SREBP2 (T); **p < 0.01, t-test, error bars = s.d. n = 3 for each assay performed.

**Figure 3:**
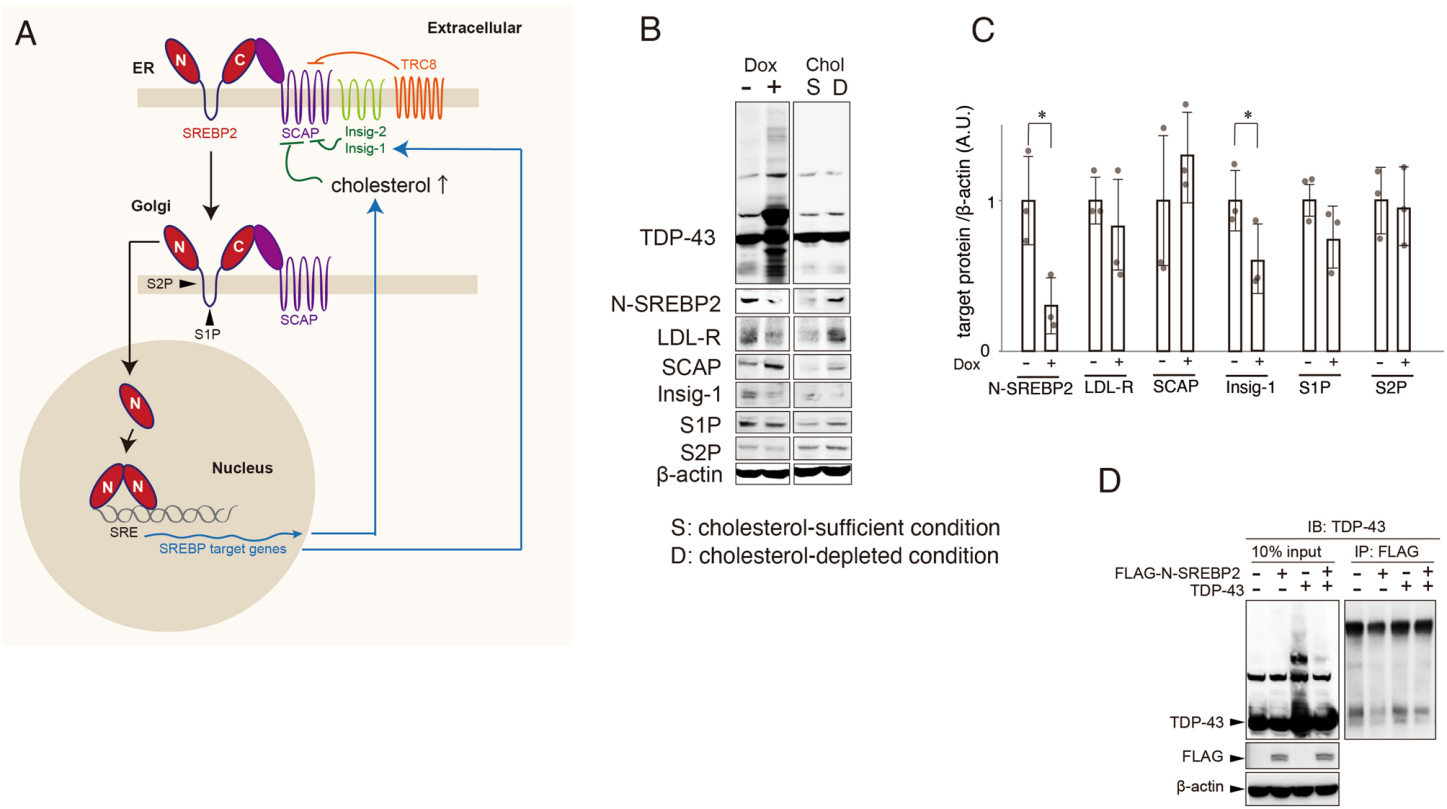
Validation of interaction between TDP-43 and SREBP2. (**A**) Post-translational modification of SREBP2. Anchor protein SCAP induces SREBP2 to translocate from endoplasmic reticulum (ER) to Golgi apparatus. SREBP2 is cleaved by S1P/S2P protease, and N-terminal SREBP2 is translocated to bind the SRE transcriptional domain of DNA for expression of target genes. (**B**) Immunoblots of proteins controlling SREBP processing pathway in TDP-43 abundant condition (Dox+) in comparison with cholesterol depleted condition. S: cholesterol-sufficient condition, D: cholesterol-depleted condition. The panels were cropped from original blots presented in Supplementary Fig. 3. (**C**) Quantification of the protein levels of cholesterol-related factors relative to β-actin between Dox− and Dox+ condition; *p < 0.05, t-test, error bars = s.d. n = 3. (**D**) HEK293T cells were transfected with or without pcDNA3.1-TDP-43 and pFLAG-NSREBP2 (1–481) and the lysates were immunoprecipitated with FLAG antibody for the following immunoblots using TDP-43 antibody.

SREBP2 is a critical transcriptional factor that regulates various enzymes in the cholesterol biosynthetic pathway by binding sterol-regulatory elements (SREs) in the promoters of cholesterol-related genes^23,24^. Western blotting revealed that TDP-43 significantly and persistently decreased the amount of the cleaved N-active form of SREBP2 (N-SREBP2) in a TDP-43 overexpression condition (Fig. 2A,B). In accordance with the suppressive effect on SREBP2 quantity, TDP-43 negatively regulated the endogenous transcriptional activity of SREBP2 as measured with a transfected SRE-luciferase construct (SRE-luc) (Fig. 2C). Elevated TDP-43 could inhibit SRE sensitivity to a decreased cholesterol level in a fashion similar to authentic sterol-regulated conditions such as cholesterol depletion by the addition of methyl-beta-cyclodextrin (MbCD) and lovastatin, HMGCoA reductase inhibitors (Fig. 2D). TDP-43 overexpression altered the amount of cholesterol (Fig. 2E). Immunocytochemistry showed that the area of nucleus-localized SREBP2 was significantly decreased compared to control when TDP-43 was overexpressed (Fig. 2F,G). It is known that SREBP2 could be modified by several post-translational mechanisms (Fig. 3A). Thus, we examined the expression levels of proteases, S1P and S2P, which cleave N-SREBP2 from the full-length SREBP2, and found that, except Insig-1, there was no remarkable difference in the two expression levels between TDP-43 abundant condition and cholesterol-depleted condition (Fig. 3B). There was also no decrement in the endoplasmic reticulum (ER) anchor protein such as SCAP protein level. The expression levels of these factors related to cholesterol biosynthesis other than N-SREBP2 and Insig-1 varied between control and TDP-43 overexpressed condition (Fig. 3C). An immunoprecipitation assay using HEK293T cells revealed that TDP-43 did not bind to N-SREBP2 (Fig. 3D). Taken together, overexpressed TDP-43 could impair cholesterol biosynthesis by inhibiting SREBP2 activity.

### Decreased cholesterol levels in spinal cord tissue of ALS model mice and in cerebrospinal fluids of ALS patients

**Figure 4:**
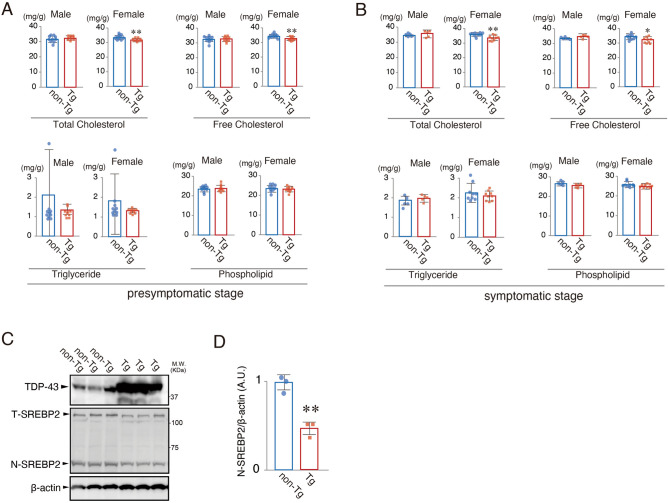
Decreased cholesterol level in spinal cord tissue of ALS model mouse. (**A**, **B**) The amounts of total cholesterol, free cholesterol, triglyceride and phospholipid per g of total spinal cord tissue were measured at 12 weeks (presymptpmatic stage, **A**) and beyond 12 weeks (symptomatic stage, **B**) after birth in A315T TDP-43 transgenic (Tg)/non-transgenic (non-Tg) male/female mice (n = 10, each group at presymptomatic stage, n = 5 male and n = 10 female at symptomatic stage); *p < 0.05, **p < 0.01, t-test, error bars = s.d. (**C**) The immunoblots of lysates of the spinal cord from non-Tg and Tg female mice. Three panels were cropped from original blots presented in Supplementary Fig. 4. (**D**) Quantification of the cleaved N-terminal form of SREBP2 relative to β-actin; **p < 0.01, t-test, error bars = s.d. n = 3.

Next, to analyze the in vivo relevance of dyslipidemia with TDP-43, we investigated the cholesterol level of spinal cord tissue from ALS model mice overexpressing A315T mutant TDP-43 (n = 10)^25^. Whole spinal cord tissues from transgenic mice or littermate control mice were fractionated to measure the amount of cholesterol per unit weight. The amounts of both total and free cholesterol were significantly decreased in the spinal cord tissue of transgenic female mice compared to their littermate controls at both pre-symptomatic stage (Fig. 4A) and symptomatic stage (Fig. 4B), suggesting that TDP-43 could impair cholesterol biosynthesis in female mice. The amount of the N-active form of SREBP2 was significantly decreased in the spinal cord tissue of transgenic female mice at the pre-symptomatic stage (Fig. 4C,D).

**Figure 5:**
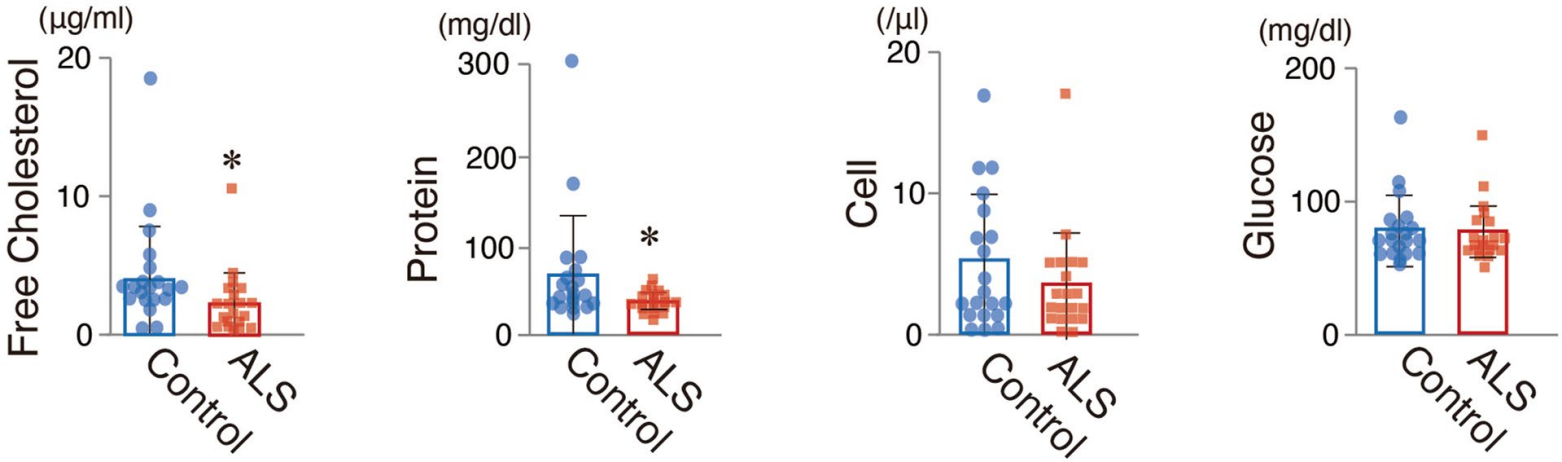
Decreased cholesterol level in the spinal fluids of ALS patients. The levels of free cholesterol, protein, cells and glucose in spinal fluids between control (*N* = 20) and ALS patients (*N* = 20); *p < 0.05, **p < 0.01, t-test, error bars = s.d.

To reveal the clinical relevance of aberrant cholesterol metabolism in the ALS pathophysiology, we examined the amount of cholesterol in the cerebrospinal fluid (CSF) of ALS patients (*N* = 20) and disease-control patients (*N* = 20) with similar characteristics (Table 1). The cholesterol amounts were significantly decreased in the CSF of ALS patients, but not in their serum (Fig. 5 and Tables 2, 3).

**Table 1:** Characteristics of study participants.

Characteristics	ALS patients	Controls	*p* value
	(*N* = 20)	(*N* = 20)	
	n (%)		
**Sex**			
Male	12 (60)	14 (70)	0.774
**Age (years)**			
Mean (S.D.)	62.2 (12.4)	61.9 (14.1)	0.570
Median (range)	64.0 (32–81)	59.0 (27–84)	
**Diabetes**			
Present	5 (25)	6 (30)	0.802
Borderline	1 (5)	0	
Unknown	14	14	
**Treatment of hyperlipidemia**			
Received	4 (20)	4 (20)	1.000

**Table 2:** Cerebrospinal fluid data between ALS patients and controls.

	ALS patients	Controls	*p* value
	(*N* = 20)	(*N* = 20)	
**Cholesterol**			
Mean (S.D.)	2.21 (2.28)	4.40 (3.94)	0.0194*
Median (range)	1.98 (0.17–10.5)	2.77 (0.50–18.9)	
**Protein**			
Mean (S.D.)	36.0 (10.3)	66.0 (64.4)	0.0263*
Median (range)	34.0 (19–59)	42.5 (26–303)	
**Glucose**			
Mean (S.D.)	76.3 (22.7)	77.7 (25.8)	0.426
Median (range)	67.5 (53–150)	69.5 (52–164)	
**Cell**			
Mean (S.D.)	3.45 (3.72)	5.16 (4.89)	0.113
Median (range)	2.5 (0–17)	3.0 (0–17)	

*p < 0.05, t-test.

**Table 3:** Serological data between ALS patients and controls.

	ALS patients	Controls	*p* value
	(*N* = 20)	(*N* = 20)	
**Cholesterol**			
Mean (S.D.)	198 (26.4)	189 (34.7)	0.251
Median (range)	195 (142–236)	179 (136–264)	
**Triglyceride**			
Mean (S.D.)	149 (69.1)	125 (81.7)	0.477
Median (range)	128.5 (60–291)	104.5(48–321)	
**HDL**			
Mean (S.D.)	51.2 (19.6)	46.1(14.4)	0.228
Median (range)	48.5 (28–107)	42.0 (28–80)	
**LDL**			
Mean (S.D.)	114 (29.3)	95.4 (37.2)	0.110
Median (range)	112 (70–167)	91.5 (35–154)	
**TP**			
Mean (S.D.)	7.00 (0.66)	7.23 (0.91)	0.169
Median (range)	6.95 (6.2–8.8)	7.30 (4.9–8.8)	
**Albumin**			
Mean (S.D.)	3.86 (0.34)	3.75 (0.54)	0.046*
Median (range)	3.90 (3.3–4.5)	3.70 (1.9–4.4)	
**A/G**			
Mean (S.D.)	1.33 (0.38)	1.16 (0.26)	0.111
Median (range)	1.35 (0.60–1.99)	1.20 (0.63–1.58)	
**BUN**			
Mean (S.D.)	14.5 (3.90)	15.8 (5.89)	0.083
Median (range)	14.0 (10–28)	15.0 (6–27)	
**Creatinine**			
Mean (S.D.)	0.59 (0.16)	0.88 (0.62)	< 0.0001*
Median (range)	0.55 (0.28–0.92)	0.65 (0.39–2.88)	
**BS**			
Mean (S.D.)	123 (44.1)	143 (82.0)	0.010*
Median (range)	106 (87–239)	119(82–420)	
**UA**			
Mean (S.D.)	5.28 (1.44)	6.69 (6.70)	< 0.0001*
Median (range)	5.3 (2.9–8.6)	5.2 (2.0–30.0)	
**IgG**			
Mean (S.D.)	1223 (521)	1351 (339)	0.104
Median (range)	1165 (707–2961)	1223 (972–2171)	
**Fe**			
Mean (S.D.)	98.5 (50.1)	92.8 (49.6)	0.987
Median (range)	98 (16–203)	94 (28–212)	
**Ferritin**			
Mean (S.D.)	209 (162)	209 (182)	0.673
Median (range)	169 (12–720)	162 (15–746)	

*p < 0.05, t-test.

## Discussion

Sterol regulation for maintaining cellular cholesterol is critical for cell growth and survival. In general, neurons may not be the main cell type biosynthesizing cholesterol in the central nervous system (CNS), as astrocytes and oligodendrocytes are the main producers and suppliers of cholesterol in the CNS^26^. TDP-43 deficiency dysregulates SREBP2 and induces demyelination in oligodendrocytes^27^, suggesting that a loss of function of TDP-43 in CNS could play an important role in ALS pathogenesis.

We found that overexpressed TDP-43 decreased the expression of genes related to cholesterol biosynthesis and impaired the transcriptional activity of SREBP2 (SI: Supplementary Fig. 1B). However, our study has several limitations, as follows.

First, the exact mechanism by which TDP-43 overexpression may regulate SREBP2 expression and functionality still remains unresolved. The N-terminal transcription domain of SREBP2 was more affected by TDP-43 overexpression, while the full-length SREBP2 was unaffected (Fig. 2A). Thus, the apparent reduced SREBF2 mRNA could not account for the observed reduction in N-terminal SREBP2. We examined the expression levels of the factors related to cholesterol biosynthesis between control and TDP-43 overexpressed condition. We found that, except for Insig-1, they varied in several experiments (Fig. 3B,C). We speculated that they may affect each other and vary in the context of the homeostasis of cholesterol biosynthesis. We examined previous TDP-43 CLIP analysis^28^ regarding whether TDP-43 could bind to the transcripts whose expression levels decreased by TDP-43 overexpression. TDP-43 binds to mRNA of SREBP2 and HMGCR but not to HMGCS1 or DHCR7. Further study is needed to elucidate how TDP-43 could alter these selected transcripts by its binding. Therefore, additional mechanisms by TDP-43 overexpression could contribute to a reduced SREBP2 mRNA level and N-terminal SREBP2 level.

Second, we found a modest yet significant difference in both total and free cholesterol in the spinal cord between control and ALS model mice, although only in female mice. A previous study revealed that male mice die of bowel obstruction due to gut dysfunction^25^, which could have masked the metabolic phenotypes in our study, and by this the cause of death.

Third, we could not identify the mechanism by which cholesterol levels were decreased in ALS patient CSF but not in the serum. It is also known that there is an increase in TDP-43 levels in ALS spinal fluid, and a negative correlation between elevated TDP-43 and cholesterol has been suggested in ALS patient CSF^29^. We speculate that abundant TDP-43 in CNS could specifically decrease the cholesterol biogenesis in ALS patient CSF.

It has been reported that the level of HMG-CoA reductase is reduced in ALS spinal cord grey matter, and that the SREBP2 expression level is lowered in ALS model mice with mutant SOD1^30^, and also that Spinocerebellar Ataxia type 2 model mice harboring TDP-43 pathology exhibit concomitant cholesterol biogenesis suppression^31^. Our observations suggest that TDP-43 impaired cholesterol biosynthesis, which is presumably involved in other neurodegenerative diseases including Alzheimer’s disease (AD) and Huntington’s disease^16,32,33^.

We speculate that a cholesterol complement may not be enough to rescue ALS MNs. Olesoxime, which has a cholesterol-like structure, has been thought to be neuroprotective for ALS MNs^34^. However, this compound failed in a phase 3 clinical trial for ALS^35^, which could underlie the requirement for a concomitant therapy that compensates for cholesterol in ALS MNs. Furthermore, as shown in Huntington’s disease, SREBP2 gene therapy may be promising for ALS^36^. Taken together, this reverse-translational study could provide a molecular basis for future ALS therapy targeting cholesterol biosynthesis.

## Supplementary Information

Supplementary Information.

## Data Availability

All data generated or analyzed during this study are included in this published article and SI.
