# Supplementary material for: TDP-43 regulates cholesterol biosynthesis by inhibiting sterol regulatory element-binding protein 2
Source: Sci Rep. 2022 May 14;12:7988. doi: 10.1038/s41598-022-12133-4 (PMC9107471; doi:10.1038/s41598-022-12133-4)
Supplement: Supplementary file 1 — Supplementary Information. [file 41598_2022_12133_MOESM1_ESM.pdf]

Supplementary Information

Title

TDP-43 regulates cholesterol biosynthesis by inhibiting sterol regulatory element-binding protein 2

Authors and affiliations

Naohiro Egawa[1,2,3], Yuishin Izumi[4], Hidefumi Suzuki[1,2,3], Itaru Tsuge[1,5], Koji Fujita[4], Hitoshi Shimano[6], Keiichi Izumikawa[7], Nobuhiro Takahashi[7], Kayoko Tsukita[1,3], Takako Enami[1,8], Masahiro Nakamura[1], Akira Watanabe[1,9], Motoko Naitoh[5], Shigehiko Suzuki[5], Tsuneyoshi Seki[10], Kazuhiro Kobayashi[10], Tatsushi Toda[10,11], Ryuji Kaji[4], Ryosuke Takahashi[2], Haruhisa Inoue[1,3,8]*

[1] Center for iPS Cell Research and Application (CiRA), Kyoto University, Kyoto, Japan

[2] Department of Neurology, Graduate School of Medicine, Kyoto University, Kyoto, Japan

[3] iPSC-based Drug Discovery and Development Team, RIKEN BioResource Research Center (BRC), Kyoto, Japan

[4] Department of Clinical Neuroscience, The University of Tokushima Graduate School, Tokushima, Japan

[5] Department of Plastic and Reconstructive Surgery, Graduate School of Medicine, Kyoto University, Kyoto, Japan

[6] Department of Endocrinology and Metabolism, Graduate School of Comprehensive Human Sciences, Tsukuba University, Ibaraki, Japan

[7]Department of Applied Biological Science, Graduate School of Agriculture, Tokyo University of Agriculture and Technology, Tokyo, Japan

[8] Medical-risk Avoidance based on iPS Cells Team, RIKEN Center for Advanced Intelligence Project (AIP), Kyoto, Japan

[9] Medical Innovation Center, Graduate School of Medicine, Kyoto University, Kyoto, Japan

[10] Division of Neurology/Molecular Brain Science, Kobe University Graduate School of Medicine, Kobe, Japan

[11] Department of Neurology, Graduate School of Medicine, The University of Tokyo, Bunkyo, Tokyo, Japan

Supplementary Figure Legends

Supplementary Figure 1. Schematic summary of main results

A. DAP (triple affinity-purification tag; biotin and FLAG tags, and N-terminal epitope tag; 6-histidine epitope tag) -TDP-43-inducible 293T Rex cells were treated with doxycycline to induce DAP-TDP-43, and the material in the lysate was used for experiments. B, TDP-43 impairs the level of SREBP2 and the related genes, leading to decrement of cholesterol biogenesis. C, The amounts of cholesterol were decreased in spinal cord tissue of ALS model mouse and in spinal fluids of ALS patients compared to control.

Supplementary Figure 2. Original blots for Figure 2A.

A. An immunoblot with dilution of 1:1,000 of TDP-43 antibody (Proteintech, #10782-1-AP).

B. An immunoblot with dilution of 1:200 of SREBP2 antibody (Cayman Chemical, #10007663).

C. An immunoblot with dilution of 1:5,000 of β-actin antibody (Sigma-Aldrich, #A5441).

Supplementary Figure 3. Original blots for Figure 3B, D.

A. An immunoblot with dilution of 1:1,000 of TDP-43 antibody (Proteintech, #10782-1-AP).
An immunoblot with dilution of 1:200 of SREBP2 antibody (Cayman Chemical, #10007663).
An immunoblot with dilution of 1:100 of LDL-R antibody (Abcam, #ab30532)
An immunoblot with dilution of 1:200 of SCAP antibody (Protein tech, #12266-1-AP)
An immunoblot with dilution of 1:500 of Insig-1 antibody (Novus Biologicals, #NB110-55244)
An immunoblot with dilution of 1:1,000 of S1P antibody (Sigma-Aldrich, #HPA040702)
An immunoblot with dilution of 1:1,000 of S2P antibody (Cell Signaling Technology, #2157S)
An immunoblot with dilution of 1:5,000 of β-actin antibody (Sigma-Aldrich, #A5441).

B. An immunoblot with dilution of 1:1,000 of TDP-43 antibody (Proteintech, #10782-1-AP).
An immunoblot with dilution of 1:1,000 of FLAG antibody (Cell Signaling Technology, #8146).
An immunoblot with dilution of 1:5,000 of β-actin antibody (Sigma-Aldrich, #A5441).

Supplementary Figure 4. Original blots for Figure 4C.

A. An immunoblot with dilution of 1:1,000 of TDP-43 antibody (Proteintech, #10782-1-AP).

B. An immunoblot with dilution of 1:200 of SREBP2 antibody (Cayman Chemical, #10007663).

C. An immunoblot with dilution of 1:5,000 of β-actin antibody (Sigma-Aldrich, #A5441).

Supplementary Methods

Immunoprecipitation (IP)

For immunoprecipitation, lysates were clarified by centrifugation at 12,000g for 10 min and then incubated overnight with Dynabeads Protein G (Thermo Fisher Scientific) bound to FLAG antibody (Cell Signaling Technology, #8146, 1:1,000). Sepharose beads were collected by brief centrifugation and washed three times with TBS buffer (50 mM Tris-HCl, 150 mM NaCl, pH7.4). Immunoprecipitated material was eluted by boiling for 3 min in 2× sample buffer (125 mM Tris-HCl, pH 6.8, 4% SDS, 20% glycerol, 0.004% BPB) and subject to SDS-PAGE for the following immunoblots.

Supplementary Figure 1

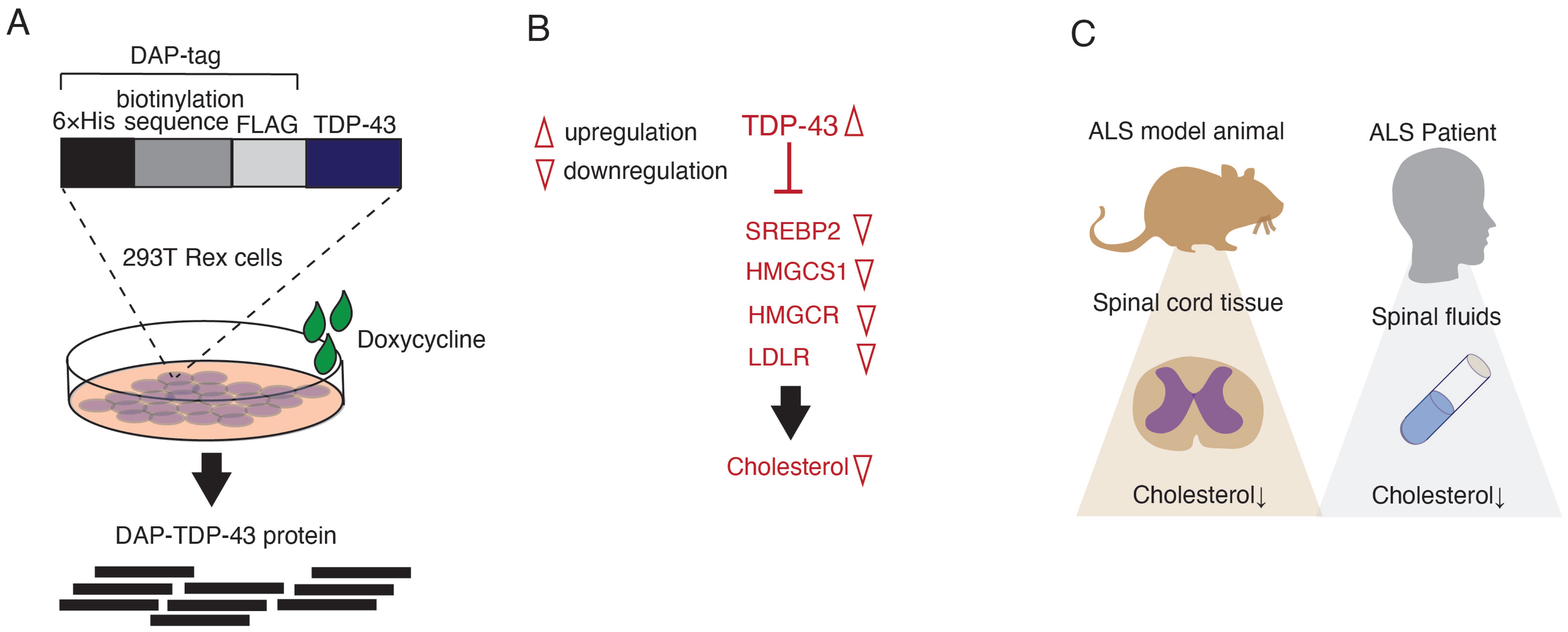

Supplementary Figure 2

A

TDP-43 (Proteintech, #10782-1-AP, 1:1,000)

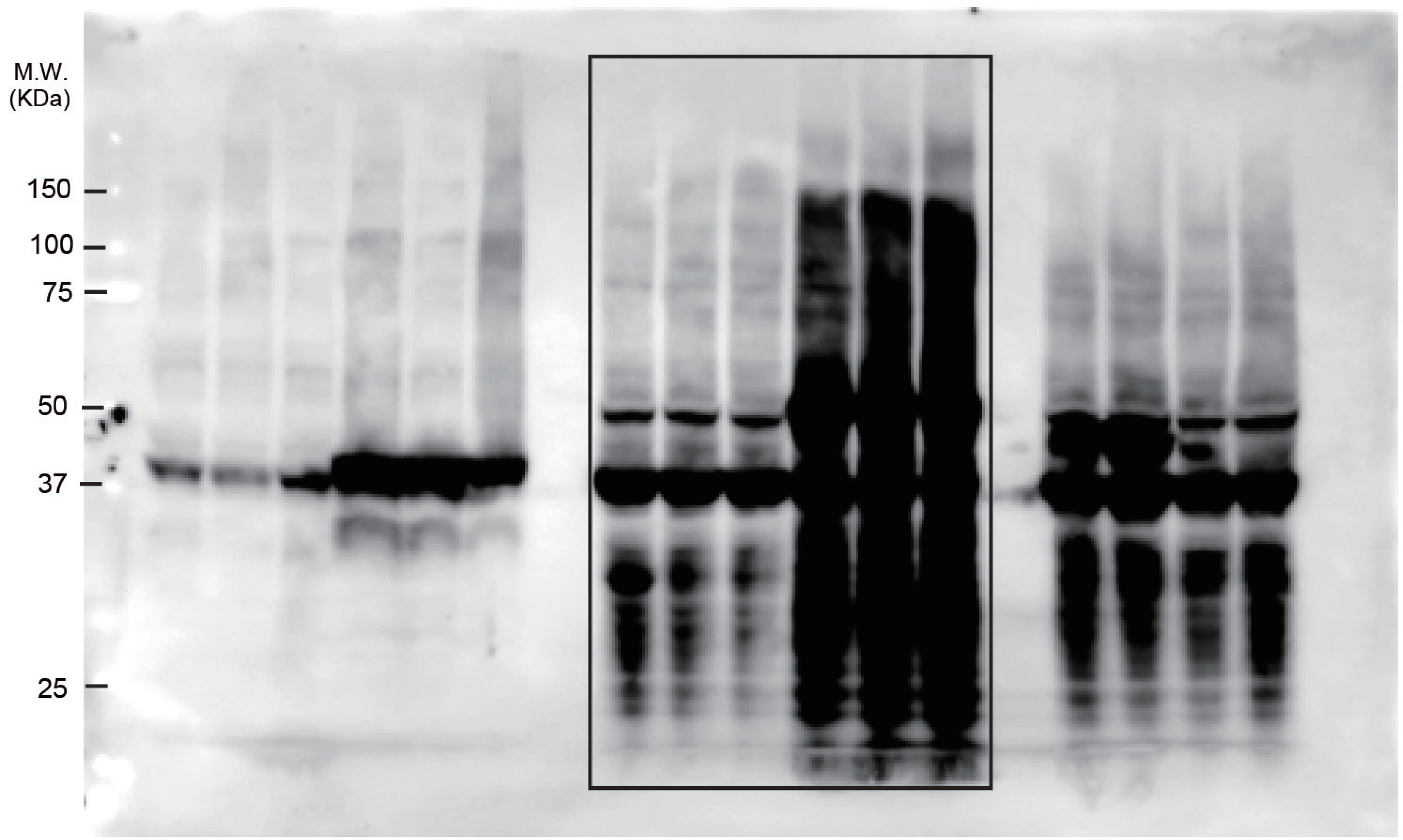

B

SREBP2 (Cayman Chemical, #10007663, 1:200)

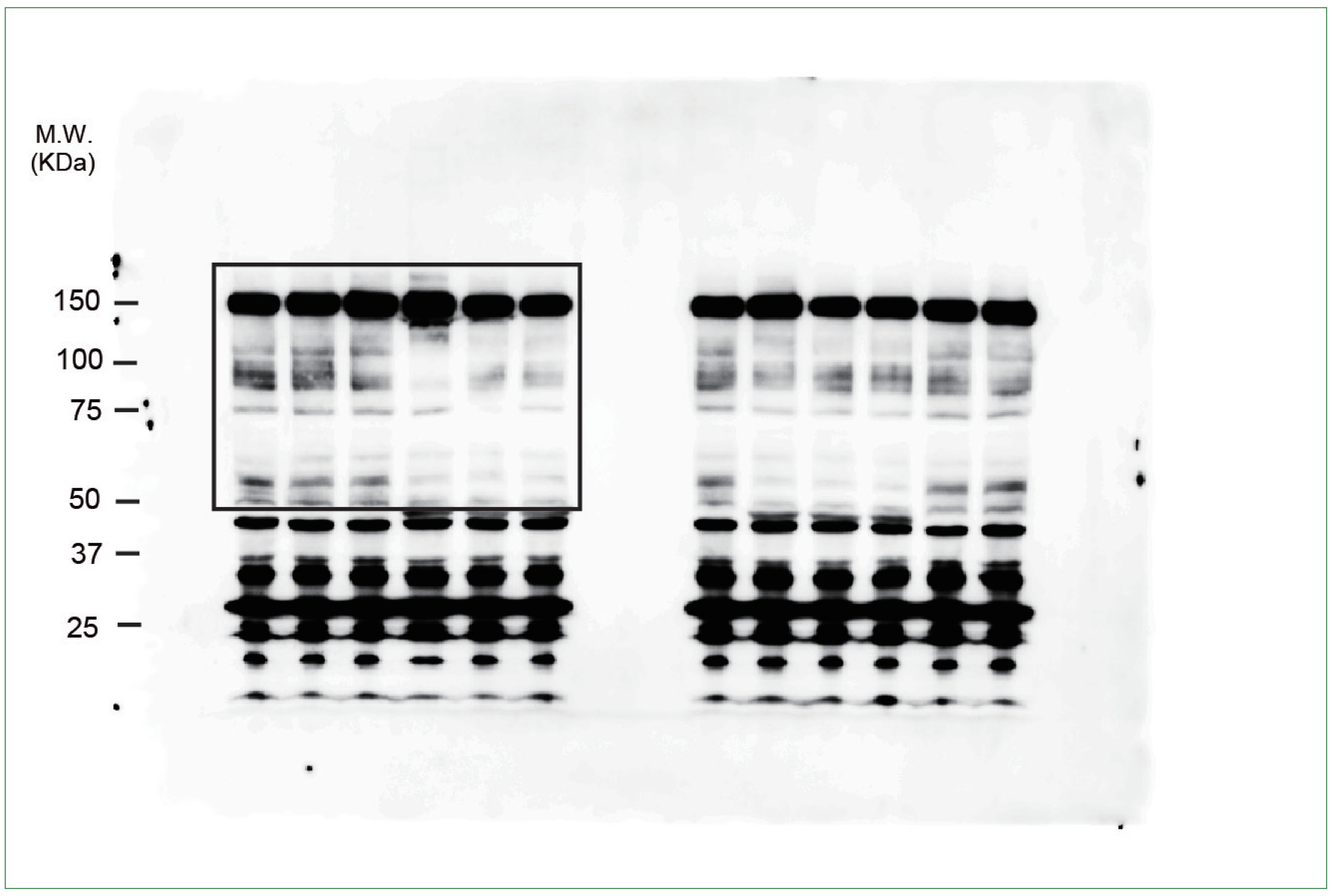

C

β-actin (Sigma-Aldrich, #A5441, 1:5,000)

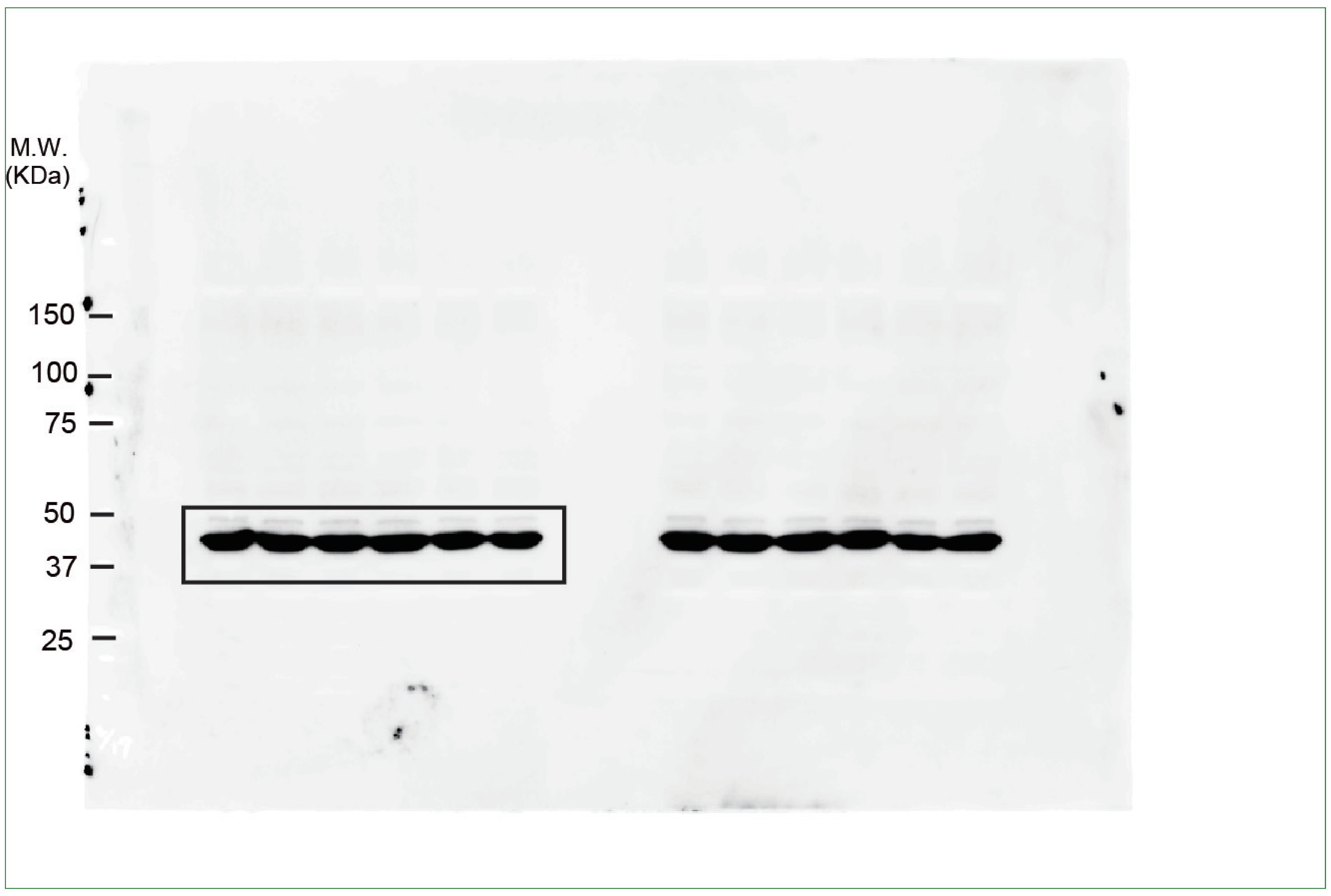

Supplementary Figure 3

A

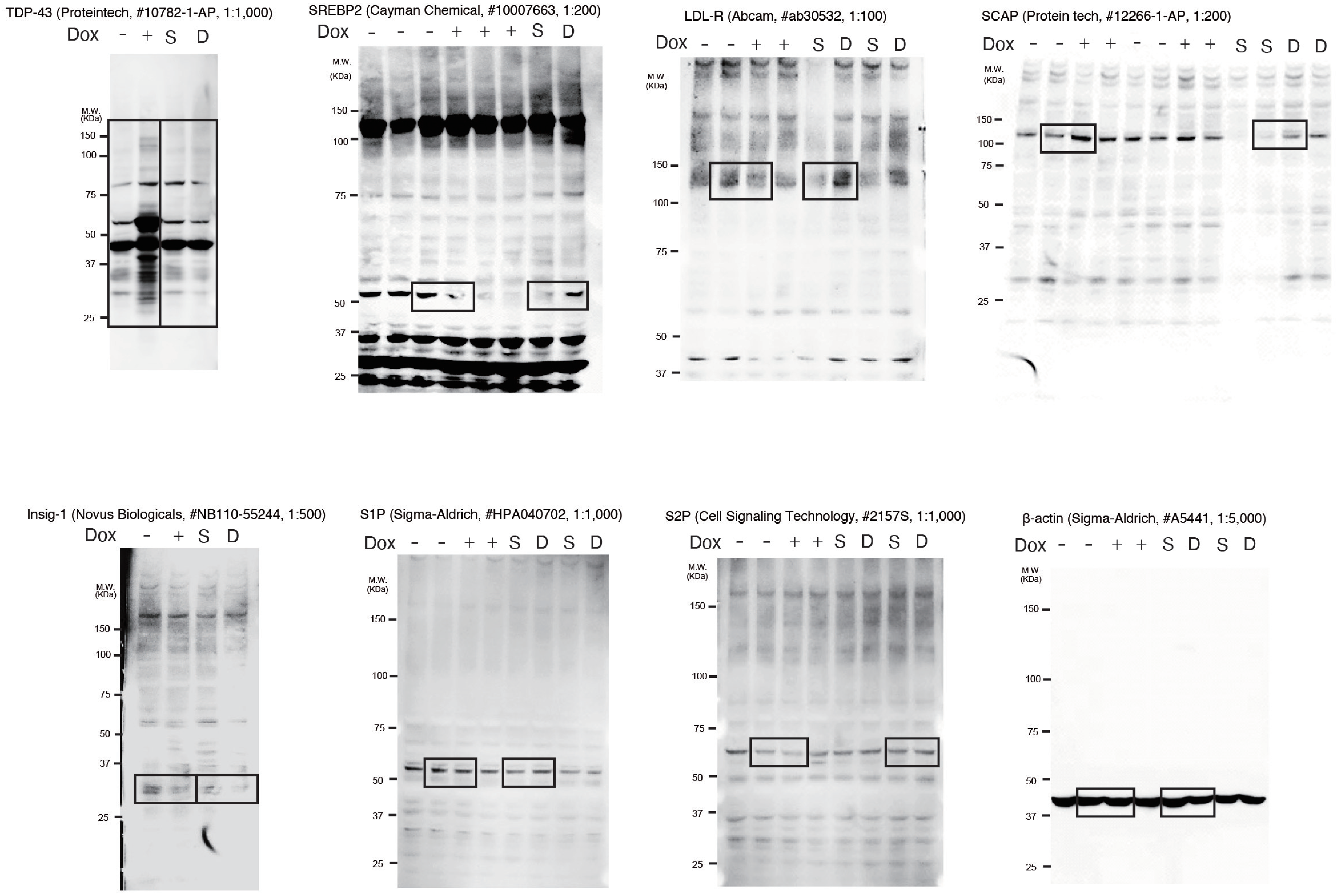

B

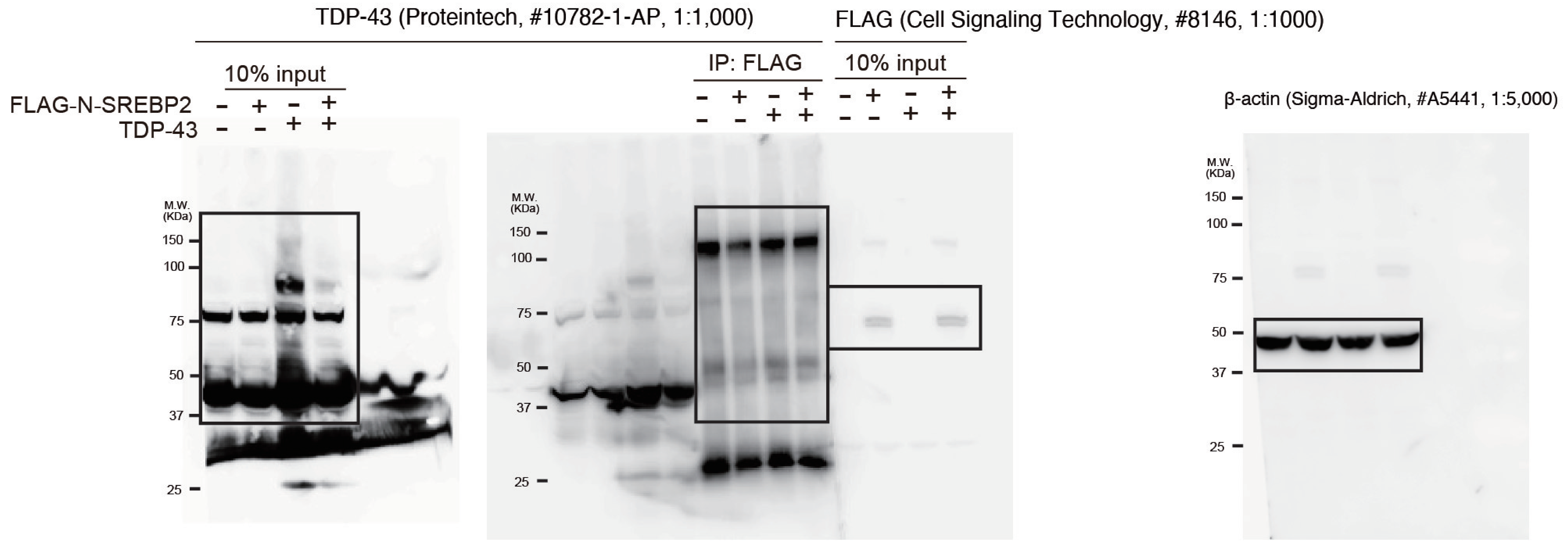

Supplementary Figure 4

A

TDP-43 (Proteintech, #10782-1-AP, 1:1,000)

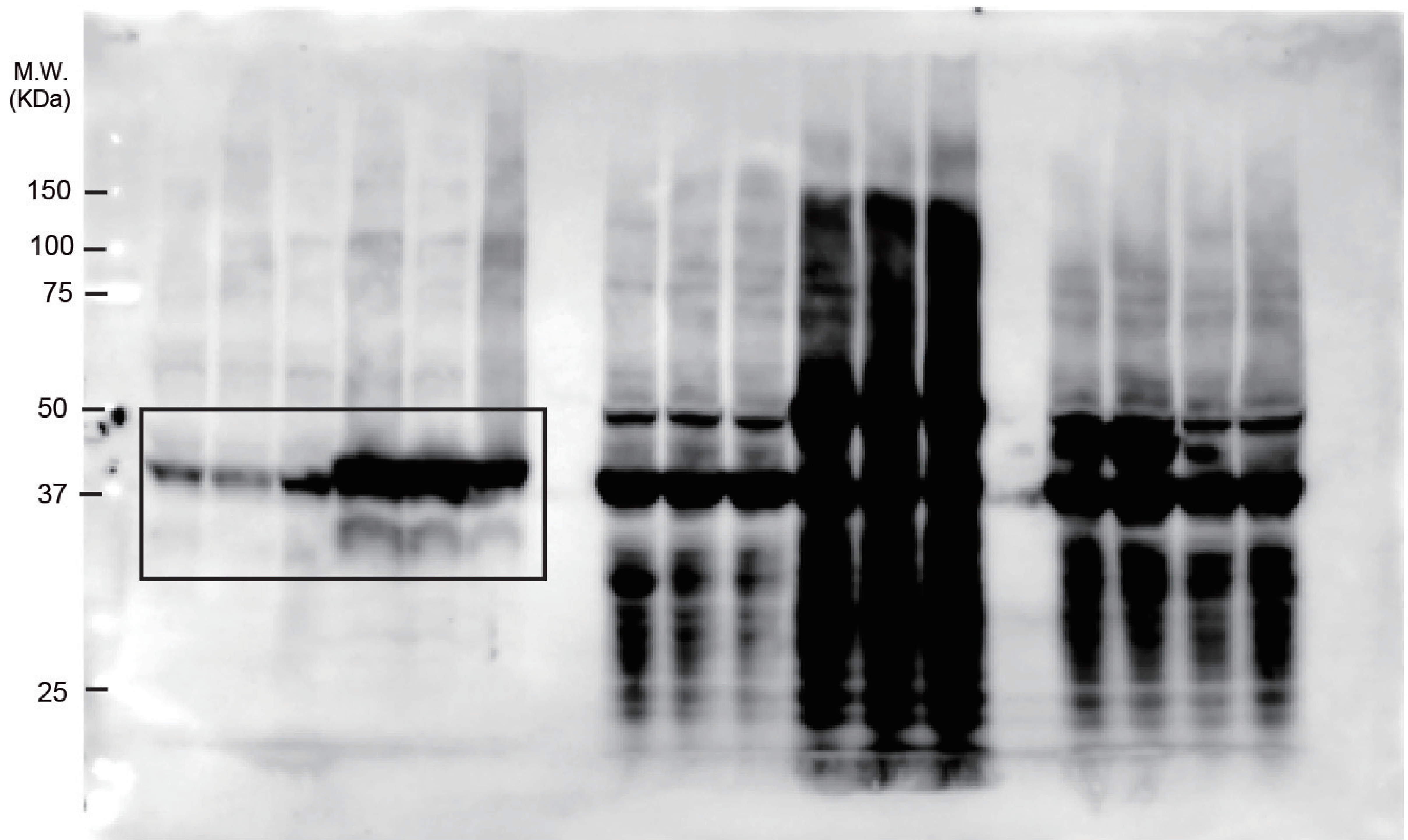

B

SREBP2 (Cayman Chemical, #10007663, 1:200)

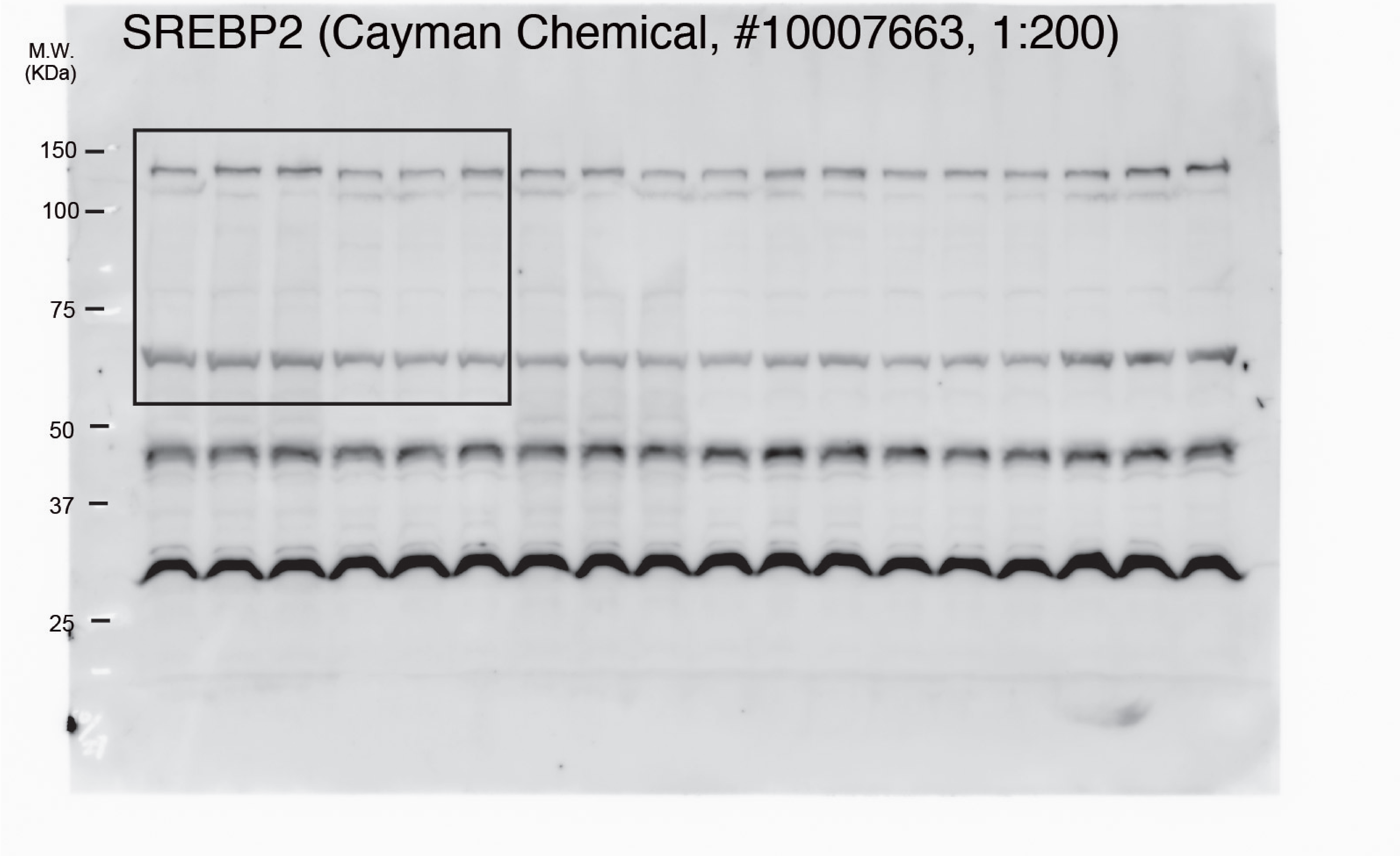

C

β-actin (Sigma-Aldrich, #A5441, 1:5,000)

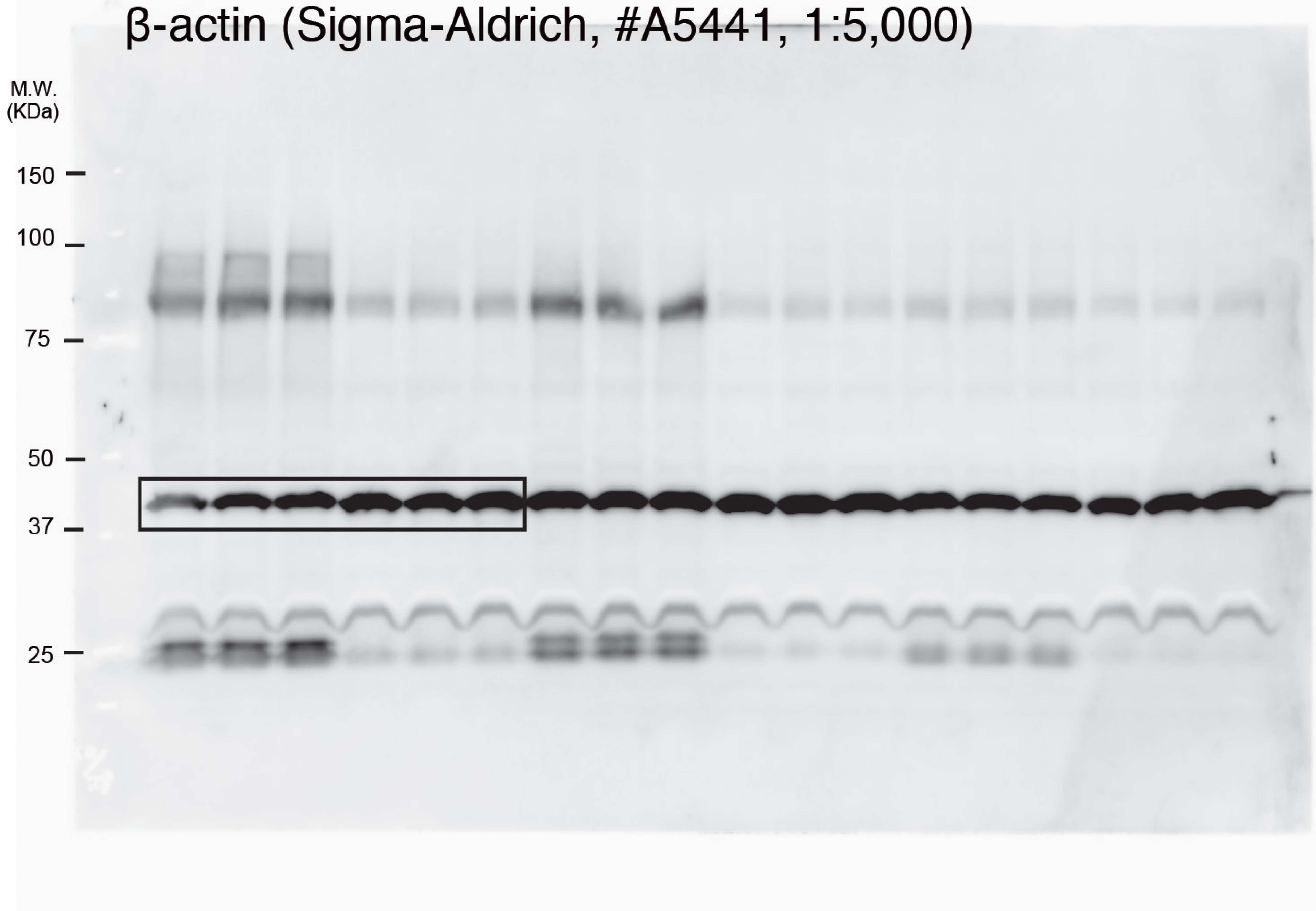

Table S1. The number of sequence reads of RNA-seq.

Sample ID	# Reads	Mapping
H1123_Rn357_DAP_TDP_43_I1, dox-	39,731,095	37,824,770
H1123_Rn358_DAP_TDP_43_I2, dox-	36,744,327	35,144,804
H1123_Rn359_DAP_TDP_43_I3, dox-	35,094,749	33,424,132
H1123_Rn360_DAP_TDP_43_I4, dox+	41,640,674	39,388,712
H1123_Rn361_DAP_TDP_43_I5, dox+	35,286,708	32,991,090
H1123_Rn362_DAP_TDP_43_I6, dox+	37,956,618	35,707,609

Table S2. Primer List

Primer list for human genes

**Primer**	**Sequence (5' to 3')**	**Applications**
GAPDH108-127F	gaaggtgaaggtcggagtca	qPCR
GAPDH287-306R	gacaagcttcccgttctcag	qPCR
SREBP2 sense	tgtgtcctcaccttcctgtgcct	qPCR
SREBP2 antisense	tccagtcaaaccagccccccaga	qPCR
HMGCS1 sense	ccccagtgtggtaaaattgg	qPCR
HMGCS1 antisense	tggcctggacttaacattcc	qPCR
SQLE sense	gctgcctgtacatcaacatc	qPCR
SQLE antisense	gaccaaggtctttgagaacat	qPCR
LDLR sense	gacgtggcgtgaacatctg	qPCR
LDLR antisense	ctggcaggcaatgctttgg	qPCR
HMGCR sense	ggaccccttgcttagatgaaa	qPCR
HMGCR antisense	ccaccaagacctattgctctg	qPCR
DHCR24 sense	gccgctctcgcttatcttcg	qPCR
DHCR24 antisense	gtcttgctaccctgctcctt	qPCR
LXRα sense	aagccctgcatgcctacgt	qPCR
LXRα antisense	tgcagacgcagtgcaaaca	qPCR
TDP-43 sense	gcgctgtacagaggacatga	qPCR
TDP-43 antisense	agttcatcccaccacccata	qPCR

Table S3. Gene ontology term under TDP-43 tet-on condition (tet-on/tet-off, F.C.<-1.2, p<0.05)

GO ID	GO ACCESSION	GO Term	corrected p-value
5754	GO:0008203	cholesterol metabolic process	0.02900672
9342	GO:0016125	sterol metabolic process	0.020572029
9343	GO:0016126	sterol biosynthetic process	0.0347833
20275	GO:0043226	organelle	0.02900672
20276	GO:0043227	membrane-bounded organelle	0.020572029

20278	GO:0043229	intracellular organelle	0.02462716
20280	GO:0043231	intracellular membrane-bounded organelle	0.019769136

Table S4. Upstream Transcriptional Regulator under TDP-43 tet-on condition

Regulator	Fold Change	Predicted State	Activation z-score	p-value of overlap
SREBP1		Inhibited	-3.644	3.63E-04
SREBP2	-2.242	Inhibited	-2.769	6.62E-09
MYCN		Inhibited	-2.372	3.70E-01
SIRT2		Inhibited	-2.236	7.49E-05
STAT5B		Inhibited	-2.219	3.92E-01
ESRRA		Inhibited	-2.156	5.28E-01
NR1H2		Inhibited	-2.144	5.93E-02
ERG		Inhibited	-2.121	1.17E-02
NR1H3		Inhibited	-2.118	1.03E-01
TBX2			-1.982	2.45E-01
STAT3			-1.964	1.00E00
PPARG			-1.928	7.76E-02
RXRA			-1.910	3.46E-01
NFkB (complex)			-1.902	1.00E00
NUPR1	1.209		-1.843	2.23E-09
CTNNB1			-1.775	1.00E00
PPARGC1B			-1.710	2.34E-03
E2F1			-1.172	4.68E-02
NR1I2			-1.080	3.20E-02
FOXO4			-0.926	5.62E-05
NKX2-3			-0.832	1.96E-02
HSF1			-0.737	1.20E-02
ATF4			-0.113	1.38E-03
NFKBIA			0.254	4.98E-02
EGR3			1.000	4.47E-02
WT1			1.231	1.34E-02
PPARA			1.253	2.93E-03
ISL1			1.969	2.38E-01
KLF4		Activated	2.219	1.13E-01
NPAT				3.49E-03
EN2				1.63E-02
HINFP				1.63E-02
ESRRB				1.64E-02
EN1				2.91E-02
SIRT1				3.05E-02

Z-score: reference of activation states of predicted transcriptional regulators in analysis in IPA®

Table S5. Gene list with significant difference between Dox ON and OFF condition (Tukey's test, q<0.05)

Gene name	Refseq_ID	log2(Expression+1;ON)	log2(Expression+1;OFF)	Var_ DoxON	Var_ DoxOFF	t.value_ DoxON:OFF	p.value_ DoxON:OFF	q.value_ DoxON:OFF
DHX9	NR_033302+NM_001357	6.14054761	7.134866779	0.0002	9.47E-05	103.8871408	9.37028E-14	1.74409E-09
PIGS	NM_033198	5.38159417	4.095550581	0.0002	0.000913	65.33898349	5.60891E-10	5.21993E-06

DENND5A	NM_015213+NM_001243254	5.12787096	3.739315505	0.0009	0.000649	61.33389341	2.28034E-09	1.4148E-05
NUP93	NM_014669+NM_001242795+NM_001242796	5.23534317	4.792856354	0.0001	7.88E-05	52.28610368	4.01653E-08	0.000186899
ENPP1	NM_006208	4.31617242	3.058282167	0.0002	0.001699	50.17188962	7.39779E-08	0.000240528
TARDBP	NM_007375	4.98602746	8.286543633	0.0004	0.012685	50.00600616	7.75356E-08	0.000240528
NCAPD3	NM_015261	5.02287051	3.487533537	0.0006	0.002881	45.20059197	2.84776E-07	0.00073556
WDR11+WDR11-AS1	NR_033850+NM_018117	5.67733364	4.152056642	0.0022	0.001274	44.79510708	3.16149E-07	0.00073556
CKAP5	NM_014756+NM_001008938	6.41126487	5.492057216	0.0013	0.000689	36.0107523	2.49282E-06	0.005155435
IKBKAP	NM_003640	4.93556857	3.105233475	8E-07	0.008532	34.31895544	3.55305E-06	0.005205295
ABI2	NM_005759	4.79692357	4.395679723	1E-04	0.000313	34.31201417	3.55812E-06	0.005205295
GABARAPL1	NM_031412	5.15571998	4.385540725	0.0002	0.001374	34.13010657	3.69326E-06	0.005205295
ZNF330	NM_014487	4.80700996	3.441187834	0.0032	0.001657	34.0012482	3.79171E-06	0.005205295
WDR41	NM_018268	5.75857468	4.16872531	0.0034	0.00335	33.61548412	4.10046E-06	0.005205295
FBL	NM_001436	8.21893466	7.733496343	0.0002	0.000441	33.50262761	4.19489E-06	0.005205295
MIR126	NR_029695	0.50260831	0	0.0007	0	32.50305979	5.11841E-06	0.005954307
CAPS2	NM_032606	1.45331037	1.278306721	3E-05	6.55E-05	30.23729103	7.89533E-06	0.00864446
STX3	NM_004177+NM_001178040	2.85301251	1.918030554	0.0018	0.001196	29.48675925	9.066E-06	0.00937475
TTF2	NM_003594	5.21390513	5.438990861	0.0002	1.1E-05	28.28550479	1.1251E-05	0.011021804
C12orf56	NM_001099676+NM_001170633	0.03182197	0	4E-06	0	27.83712022	1.21752E-05	0.011174492
C7orf41	NM_152793	3.70768624	2.783701376	0.0028	0.000719	27.04228526	1.39741E-05	0.011174492
SF3B3	NM_012426	5.40173809	4.854004546	0.0009	0.000293	26.96405329	1.41629E-05	0.011174492
COL4A5	NM_000495+NM_033380	2.22795429	1.672809189	0.0006	0.000653	26.90860274	1.42981E-05	0.011174492
LOC643387	NR_026923	1.54333962	0.23984717	0.0029	0.004429	26.39084599	1.56146E-05	0.011174492
HADHB	NM_000183	5.17325142	3.821887969	0.003	0.004946	26.27018753	1.5936E-05	0.011174492
PIGC	NM_153747+NM_002642	5.05330556	4.147665795	0.0023	0.001313	26.17624699	1.61901E-05	0.011174492
MTCH2	NM_014342	5.00131626	4.030730743	0.0027	0.001504	25.99685351	1.66853E-05	0.011174492
TXNRD3+TXNRD3NB	NM_001039783+NM_001173513+NM_052883	3.88873075	4.378021226	0.0008	0.000281	25.95238107	1.68101E-05	0.011174492
HARS+HARS2	NM_002109+NM_001258040+NM_001258042+NM_001258041+NM_012208	6.30492256	5.881407128	1E-04	0.000722	25.61793421	1.77747E-05	0.011232556
JHDM1D	NM_030647	3.33576381	2.224939638	0.0002	0.005607	25.33313945	1.86338E-05	0.011232556
PRPS1	NM_001204402+NM_002764	6.23828798	5.844449501	0.0006	0.000143	25.30913523	1.87079E-05	0.011232556
MDFIC	NM_199072+NM_001166346+NM_001166345	3.5417323	2.541660983	0.0031	0.001817	24.78954779	2.03757E-05	0.011851671
MSH4	NM_002440	0.09034095	1.306180144	0.0006	0.006868	24.2986966	2.20719E-05	0.012397316
SLC6A13	NM_016615+NM_001190997+NM_001243392	0.11312595	2.01812809	0.0015	0.017539	23.93608287	2.34059E-05	0.012397316
POU2F3	NM_001244682+NM_014352	0.03535624	0	7E-06	0	23.29927139	2.59311E-05	0.012397316
ATP9B	NM_198531	2.58914007	1.788049021	0.0021	0.001418	23.29717821	2.59398E-05	0.012397316
SLC37A3	NM_032295+NM_207113	4.51226485	3.819229936	0.0009	0.001856	23.04918174	2.69916E-05	0.012397316
FAM149B1	NM_173348	3.83348149	3.48507926	0.0006	0.000128	22.93675832	2.74818E-05	0.012397316
RNF114	NM_018683	5.534174	4.649768084	0.0027	0.001758	22.83634571	2.7927E-05	0.012397316
DDX55	NM_020936	4.69908786	4.005130081	0.0026	0.000319	22.3823141	3.0031E-05	0.012397316
GLOD5	NM_001080489	0	0.067201057	0	2.71E-05	22.34494971	3.02111E-05	0.012397316
PTGDS	NM_000954	0	0.061124128	0	2.25E-05	22.29777099	3.044E-05	0.012397316
ASB17	NR_026546+NM_080868	0	0.047718956	0	1.39E-05	22.19392664	3.09502E-05	0.012397316
ACTL7A	NM_006687	0	0.034954197	0	7.51E-06	22.09533469	3.14428E-05	0.012397316
GJA4	NM_002060	0	0.030924733	0	5.89E-06	22.06427111	3.15996E-05	0.012397316

IL17A	NM_002190	0	0.027019025	0	4.51E-06	22.0341886	3.17523E-05	0.012397316
PNLIPRP3	NM_001011709	0	0.02180238	0	2.95E-06	21.99405052	3.19573E-05	0.012397316
CCDC83	NM_173556	0	0.021460933	0	2.86E-06	21.991425	3.19707E-05	0.012397316
SUV420H1	NM_017635+NM_016028	3.98408387	4.457934588	0.0013	0.00013	21.50819738	3.45522E-05	0.013124891
SLC6A4	NM_001045	0.05023635	0.377416637	8E-05	0.000643	21.12853165	3.67395E-05	0.01367666
HIST1H2AH	NM_080596	8.01255284	8.54128127	0.0002	0.001749	20.87901761	3.82617E-05	0.013775822
STT3B	NM_178862	5.9877224	4.78172353	0.0024	0.007788	20.72448849	3.92408E-05	0.013775822
ARHGEF9	NM_001173479+NM_015185+NM_001173480	1.9657975	0.818195439	0.0073	0.002198	20.35214971	4.17235E-05	0.013775822
OXCT1	NM_000436	4.97041871	3.996036486	0.0062	0.000913	20.07760035	4.36761E-05	0.013775822
PRKAG1	NM_001206710+NM_002733+NM_001206709	5.74851026	5.246340717	0.0002	0.001687	20.02261395	4.40805E-05	0.013775822
SMARCA1	NM_003069+NM_139035	6.19232945	5.584327865	0.0022	0.000632	19.86618095	4.52569E-05	0.013775822
NUDT18	NM_024815	2.97977854	3.50226002	0.0007	0.001432	19.81629276	4.56403E-05	0.013775822
PFKM+SENP1	NM_001267595+NR_051991+NR_051992+NM_001267594+NM_001166686+NM_000289+NM_001166688+NM_001166687	5.42611834	5.297874139	6E-05	6.5E-05	19.80791326	4.57051E-05	0.013775822
SCAMP1	NM_004866	5.17965767	3.795506658	0.0101	0.004605	19.77605325	4.59526E-05	0.013775822
SLC25A38	NM_017875	4.73221006	4.955033936	0.0002	0.000138	19.69854003	4.65618E-05	0.013775822
NUP85	NM_024844	5.63983279	4.5626457	0.0004	0.008563	19.66385144	4.68377E-05	0.013775822
MAP3K15+PDHA1	NM_001173456+NM_000284+NM_001173455+NM_001173454+NM_001001671	6.53917091	6.220914006	0.0004	0.000441	19.59606311	4.73831E-05	0.013775822
PHF20	NM_016436	4.34261037	3.673585632	0.0001	0.003419	19.51600928	4.80376E-05	0.013775822
VARS2	NM_020442+NM_001167733+NM_001167734	4.53245388	3.179114743	0.0088	0.005888	19.31255888	4.97546E-05	0.013775822
GAPVD1	NM_015635	4.79592965	4.582613972	0.0002	0.000164	19.29313069	4.99227E-05	0.013775822
AIFM1	NM_001130846+NM_001130847+NM_145813+NM_145812+NM_004208	5.37993863	3.786236174	0.0114	0.009095	19.2874068	4.99724E-05	0.013775822
GPR179	NM_001004334	0.03142292	0.144274427	2E-05	8.48E-05	19.20314971	5.07111E-05	0.013775822
PROSC	NM_007198	3.97985177	4.36674763	0.0004	0.000821	19.19391342	5.0793E-05	0.013775822
LAMC1	NM_002293	5.46082639	4.685165242	0.0022	0.002676	19.16300926	5.10682E-05	0.013775822
TAPT1	NM_153365	3.7056191	2.80211437	0.0061	0.000757	18.96633324	5.28665E-05	0.013871329
SLTM	NM_001013843+NM_024755	4.64330165	3.575034395	0.0014	0.00815	18.9613998	5.29127E-05	0.013871329
TMEM245	NM_032012	4.39475405	3.766927295	0.0033	5.81E-05	18.75439771	5.49007E-05	0.014121217
CHD1L	NM_004284+NR_046070+NM_001256336+NM_001256337+NM_001256338+NM_024568	5.76681014	5.207384154	0.0019	0.000754	18.705666	5.53833E-05	0.014121217
NSG1+STX18	NM_014392+NM_001040101+NM_016930	4.65680365	3.828194199	0.0033	0.002691	18.52724959	5.72003E-05	0.014387433
CLN5	NM_006493	3.37964929	3.71455232	0.0007	0.00028	18.41161602	5.84218E-05	0.014469346
FAM91A2+LINC00623	NR_046135+NR_046136+NR_046137+NR_046138+NR_046139+NR_024510	3.0006719	3.318947136	0.0007	0.000161	18.26529992	6.00195E-05	0.014469346
TBC1D9B	NM_015043+NM_198868	5.20074133	4.170978339	0.003	0.006674	18.15630656	6.12492E-05	0.014469346
LAMA1	NM_005559	3.37894303	2.316357058	0.0034	0.006915	18.14061764	6.14291E-05	0.014469346
G3BP1	NM_005754+NM_198395	7.01267072	7.234427258	0.0004	6.63E-05	18.09743873	6.1928E-05	0.014469346
GABRB3	NM_001191320+NM_000814+NM_001191321+NM_021912	3.76954283	2.885812787	0.0016	0.005704	17.97159132	6.34147E-05	0.014469346

ELL3+HYPK+MIR1282+PDIA3+SERF2+SERF2-C15ORF63+SERINC4	NM_005313+NM_025165+NM_001199877+NM_001018108+NM_001199875+NM_001199876+NR_037673+NR_037672+NM_001199878+NR_031695+NM_001258032+NM_001258031+NM_001199885+NM_016400	8.11390355	7.559626872	0.0026	0.0003	17.72255898	6.65075E-05	0.014469346
SLC47A1	NM_018242	3.97297832	3.241925329	4E-05	0.005082	17.69894742	6.68117E-05	0.014469346
FUBP3	NM_003934	4.7261633	3.994090116	0.0013	0.003843	17.69212185	6.69E-05	0.014469346
DHX15	NM_001358	6.89283714	6.104471513	0.0049	0.001065	17.63933371	6.75884E-05	0.014469346
VWA9	NM_001207059+NM_001207058+NR_045106+NR_045105+NR_045104+NR_045107+NR_045901	5.66797368	5.927987955	0.0006	6.43E-05	17.63392411	6.76596E-05	0.014469346
DCTN2+MBD6	NM_052897+NM_006400+NM_001261413+NM_001261412	6.04008069	5.48458878	0.0003	0.002733	17.59755349	6.81404E-05	0.014469346
INTS2	NR_026641+NM_020748	4.3319596	3.994132616	0.0004	0.000688	17.58992198	6.82419E-05	0.014469346
STK38	NM_007271	3.2463441	2.33932415	1E-05	0.008003	17.54903363	6.87895E-05	0.014469346
IGF2R	NM_000876	3.50850241	1.75561142	0.0092	0.020877	17.51965726	6.91867E-05	0.014469346
GTF2IP1+LOC100093631	NR_003580+NR_002206	6.18693121	6.47079727	0.0006	0.000205	17.41071978	7.06883E-05	0.014502332
POLA1	NM_016937	5.84883422	5.000806488	0.0031	0.00406	17.39457057	7.09149E-05	0.014502332
IDH3B	NM_006899+NM_174855+NM_001258384	6.66430164	6.140518124	0.0006	0.002127	17.27389255	7.26406E-05	0.014502332
NARG2	NM_024611+NM_001018089	4.2464347	4.631770617	0.001	0.000509	17.23177426	7.3257E-05	0.014502332
SPATA6	NM_019073	1.83910094	2.09319045	0.0002	0.000433	17.1896519	7.38808E-05	0.014502332
SLC9A6	NM_001177651+NM_006359+NM_001042537	3.71305177	3.858192874	2E-05	0.000199	17.18036883	7.40193E-05	0.014502332
CPNE1+RBM12	NM_152925+NM_003915+NM_001198863+NM_152928+NM_152927+NM_152926+NR_037188+NM_006047+NM_001198840+NM_152838+NM_001198838	6.49513129	7.096000563	0.001	0.002819	16.90254424	7.83407E-05	0.015189127
LAMB1	NM_002291	5.07609629	4.347048062	0.0025	0.003318	16.60883374	8.33092E-05	0.015985918
DKC1	NM_001363+NM_001142463	6.05596353	4.685696219	0.0017	0.018918	16.52933783	8.47307E-05	0.016092629
TBX15	NM_152380	0.14697258	1.068896079	0.0069	0.002474	16.48194589	8.55945E-05	0.016092629
JRKL	NM_003772+NM_001261833	2.8110255	2.061187649	0.0028	0.003512	16.34061143	8.82459E-05	0.0163142
RMI2	NM_152308	4.90943643	4.330430488	0.0001	0.003717	16.21582917	9.06843E-05	0.0163142
DDO	NM_003649+NM_004032	0	0.031614334	0	1.14E-05	16.21536633	9.06935E-05	0.0163142
ACTR8	NM_022899	3.86181963	3.124044685	0.0052	0.001033	16.1699634	9.16045E-05	0.0163142
FAM228B	NM_001145710	2.76402347	3.931509489	0.0009	0.014837	16.11460716	9.27327E-05	0.0163142
TTLL1	NR_027779+NM_012263	2.45885301	3.30642619	0.001	0.007305	16.08623112	9.33186E-05	0.0163142
CTNNBL1	NM_030877	4.80508913	4.195093785	4E-05	0.004355	15.9363225	9.65022E-05	0.0163142
TRA2A	NM_013293	4.97400792	5.923012496	0.0026	0.008007	15.93009048	9.66378E-05	0.0163142
PIK3C2A	NM_002645	4.23504241	3.238329128	0.0013	0.010555	15.86011861	9.81792E-05	0.0163142
SNHG16	NR_038111+NR_038110+NR_038108+NR_038109	7.86658626	7.338001662	0.0002	0.003128	15.83621472	9.87137E-05	0.0163142
HMGCS1	NM_002130+NM_001098272	5.68078012	4.599970428	0.0084	0.005562	15.82861205	9.88845E-05	0.0163142
CDC42BPB	NM_006035	3.99970256	3.228870371	6E-05	0.007061	15.81876242	9.91065E-05	0.0163142
PHF3	NM_015153	5.03402372	4.374050631	0.0031	0.002141	15.80687093	9.93753E-05	0.0163142
CRIM1	NM_016441	3.75607388	2.483691854	0.0007	0.018891	15.72873827	0.000101168	0.0163142
TTC37	NM_014639	6.26864719	5.306335269	0.0068	0.004473	15.72259579	0.000101311	0.0163142
FANCG	NM_004629	4.40107895	3.875261162	0.0009	0.002475	15.72183599	0.000101328	0.0163142
GTF2IP1+LOC100093631	NR_002206+NR_003580	6.22057488	6.515592632	0.0008	0.000214	15.70704536	0.000101673	0.0163142

SREBF2	NM_004599	3.95470196	2.664535228	0.0086	0.011924	15.59244397	0.000104404	0.01648623
CDCA5	NM_080668	5.18990717	4.714423528	0.0023	0.000477	15.57287776	0.000104881	0.01648623
IGSF3	NM_001542+NM_001007237	3.5732734	2.57611078	0.0049	0.007511	15.47202973	0.000107385	0.01648623
GLB1L2	NM_138342	4.00101072	3.530234386	0.0003	0.002478	15.45443846	0.00010783	0.01648623
GJB7	NM_198568	0.83880837	0.219915573	0.0032	0.001592	15.40988646	0.000108969	0.01648623
PHF16	NM_001077445+NM_014735	3.88053193	2.494888082	0.0068	0.017522	15.39362546	0.000109389	0.01648623
PM20D2	NM_001010853	4.31981123	4.666889142	0.0015	2.35E-05	15.37715285	0.000109817	0.01648623
CD99L2	NM_031462+NM_001184808+NM_001242614+NM_134446+NM_134445	4.44294258	4.241173775	0.0004	0.000102	15.3766036	0.000109831	0.01648623
XPC	NM_001145769+NR_027299+NM_004628	4.66538518	4.171702531	0.0021	0.001036	15.3198335	0.000111324	0.016576553
TBC1D8	NM_001102426	2.41722041	2.053237517	0.0003	0.001437	15.22076844	0.000113996	0.016839713
FIG4	NM_014845	3.94707999	3.325927517	0.0029	0.002138	15.13702339	0.000116324	0.017048353
CLK1	NM_004071+NM_001162407+NR_027856+NR_027855	4.56126343	3.881266959	0.0048	0.001392	14.97485982	0.000121022	0.017598325
ROBO2	NM_002942+NM_001128929	1.61093851	1.791740829	0.0001	0.000301	14.89630911	0.000123392	0.017770675
MGEA5	NM_012215+NM_001142434	5.43766376	5.057663801	0.0018	0.000188	14.87267831	0.000124117	0.017770675
HIST1H1D	NM_005320	7.31672243	7.895435454	0.0008	0.003746	14.80961068	0.000126081	0.017914113
AP2M1	NM_001025205+NM_004068	6.56824524	5.89772475	0.0038	0.002501	14.6639457	0.000130783	0.018441359
ELP2	NM_018255+NM_001242878+NM_001242876+NM_001242877+NM_001242879+NR_040110+NM_001242875	5.19296646	4.692933233	0.0021	0.001362	14.62447447	0.000132098	0.018486725
BLMH	NM_000386	5.54798734	5.114501645	0.0025	0.000109	14.56309112	0.000134178	0.018498046
C1orf101	NM_173807+NM_001242340+NM_001130957	0.09455897	0.281992972	0.0003	0.000184	14.51812635	0.000135731	0.018498046
HS6ST2	NM_001077188+NM_147175	4.31767968	4.647033918	0.0004	0.001129	14.51605692	0.000135803	0.018498046
BBS7	NM_176824+NM_018190	4.98857283	5.284653071	0.0002	0.001055	14.50337828	0.000136245	0.018498046
GTF2I	NM_001163636+NM_033001+NM_001518+NM_033000+NM_032999	6.21474253	6.539838064	0.0001	0.001414	14.44703411	0.000138235	0.018498046
CDC23+KIF20A	NM_005733+NM_004661	6.65396258	6.075066045	0.0004	0.004436	14.4088603	0.000139606	0.018498046
SEL1L3	NM_015187	3.58775939	3.266860393	0.0002	0.00128	14.40199298	0.000139854	0.018498046
TTL	NM_153712	4.57582114	4.43897317	0.0002	0.000111	14.39442114	0.000140129	0.018498046
DDX17+KDELR3	NM_006855+NM_016657+NM_006386+NM_001098504	6.87141418	7.187354048	0.0006	0.000846	14.28035194	0.000144357	0.018921905
ZNF688	NM_145271+NM_001024683	2.14233632	2.660109335	0.0008	0.003224	14.19218093	0.000147742	0.01923028
ESPL1	NM_012291	4.01089506	3.514162324	0.001	0.002747	14.07804034	0.000152285	0.019526207
PRSS23	NM_007173	4.20989486	4.786482533	0.004	0.001107	14.04257043	0.000153735	0.019526207
BRAP	NM_006768	4.09783066	3.298362587	0.0027	0.007059	14.03313142	0.000154124	0.019526207
MCOLN2	NM_153259	3.63560453	3.02039916	0.0057	3.8E-05	14.03098777	0.000154212	0.019526207
LOC653712	NR_034179	1.21579931	1.538379723	0.0008	0.000771	13.97239015	0.000156658	0.019701833
C20orf72	NM_052865	5.09726627	4.436625138	0.0023	0.004412	13.92332573	0.000158745	0.019779607
TSSC1	NM_003310	4.48937102	4.682350622	0.0003	0.000278	13.90807872	0.000159402	0.019779607
NUDT10	NM_153183	3.38793114	1.722031157	0.0005	0.042974	13.84491449	0.000162159	0.019988496
LOC100289187	NM_001195543+NM_001195542+NM_001195541	2.79996768	3.858428232	0.0103	0.007404	13.77787396	0.000165155	0.020051216
KLHL23+PHOSPHO2+PHOSPHO2-KLHL23	NM_001199288+NM_001199286+NM_001199287+NM_001199290+NM_001199285+NM_001008489+NM_144711	5.51191857	4.832322956	0.006	0.001276	13.77583725	0.000165247	0.020051216
SORL1	NM_003105	1.62541947	0.72466391	0.0061	0.006752	13.73714708	0.000167011	0.020051216
CNOT6	NM_015455	3.54812772	2.677358535	0.0086	0.003463	13.72705303	0.000167475	0.020051216
ANKRD18B	NM_001244752	2.35875908	2.516550185	8E-05	0.000315	13.71354197	0.000168099	0.020051216

ABCB7	NM_001271696+NM_001271697+NM_001271698+NM_001271699+NM_004299	5.3754255	4.672034232	0.0002	0.007751	13.68076825	0.000169626	0.020051216
LZIC	NM_032368	4.4333107	5.103843062	0.0013	0.006006	13.62874307	0.000172087	0.020051216
METAP1	NM_015143	4.74171477	4.384531166	0.0002	0.00186	13.61120584	0.000172928	0.020051216
C11orf1+FDXACB1	NR_038364+NM_138378+NM_022761	4.17923273	4.854530354	0.0069	0.000448	13.60401379	0.000173274	0.020051216
SDF2	NR_045585+NM_006923	4.4047637	3.554030195	0.0002	0.011587	13.60056108	0.00017344	0.020051216
KLHL7	NM_001172428+NR_033329+NM_001031710+NR_033328+NM_018846	4.72658004	4.286807147	0.0018	0.001353	13.50124024	0.000178322	0.020488293
GCSHP3	NR_033248	2.37384487	1.132527483	0.006	0.019459	13.46398024	0.0001802	0.020577027
RCCD1	NM_001017919+NM_033544	4.0617448	3.295362534	0.0038	0.00604	13.35822086	0.000185673	0.021005573
ITGB1	NM_133376+NM_002211+NM_033668	7.36244331	6.831925043	0.0006	0.004124	13.32056862	0.000187674	0.021005573
SELO	NM_031454	3.30605545	2.794263327	0.0015	0.002929	13.31059665	0.000188209	0.021005573
DTYMK	NR_033255+NM_001165031+NM_012145	5.87277725	6.184458492	0.0006	0.001094	13.30580021	0.000188467	0.021005573
ZNF883	NM_001101338	3.33449498	3.751525	0.0018	0.001227	13.20100327	0.000194219	0.021517812
XRCC5	NM_021141	7.35370974	7.02295946	0.0015	0.000366	13.14961799	0.000197123	0.021710344
HMGCR	NM_001130996+NM_000859	4.74398611	4.179012701	0.0044	0.001152	13.0853775	0.000200834	0.02198893
DISC1+DISC2+TSNAX+TSNAX-DISC1	NR_028399+NR_028395+NR_028397+NR_028396+NR_028394+NR_028393+NR_028400+NR_028398+NM_005999+NM_001164554+NM_001164538+NM_001164549+NM_001164539+NM_018662+NM_001164553+NM_001164552+NM_001164551+NM_001164550+NM_001164555+NM_001164537+NM_001164548+NM_001164545+NM_001164547+NM_001164546+NM_001164540+NM_001164544+NM_001164542+NM_001164541+NM_001012958+NM_001012957+NM_001012959+NM_001164556+NR_002227	5.54931987	4.26856918	0.0125	0.016411	13.0388294	0.00020358	0.022159217
FLJ23152	NM_001190766	1.44369739	1.989757895	0.0003	0.005023	12.95092995	0.000208899	0.02251021
C1orf95	NM_001003665	0.60861989	0.843907414	0.0008	0.000233	12.93492072	0.000209887	0.02251021
HOMER1	NM_004272	4.68650117	4.90584379	0.0006	0.000229	12.9117875	0.000211326	0.02251021
PSPH	NM_004577	4.48467195	4.786469405	0.0001	0.001536	12.90673865	0.000211642	0.02251021
SERPINE2	NM_001136530+NM_001136528+NM_006216+NR_073116	4.41347079	3.983651798	0.0013	0.002039	12.81788047	0.000217299	0.022980625
LOC728739	NR_037629	0.7092161	1.748933512	0.0151	0.004786	12.75825135	0.000221206	0.023158146
AP3M2	NM_001134296+NM_006803	5.00841717	4.570896112	0.0003	0.003263	12.75432574	0.000221466	0.023158146
MAOA	NM_001270458+NM_000240	3.13505657	2.464846532	0.0074	0.000906	12.72637808	0.000223331	0.023222709
ARL14EP	NM_152316	4.20801492	3.424505325	0.0094	0.002036	12.69393107	0.000225522	0.023320242
TBXAS1	NM_001166254+NM_001130966+NM_001166253+NR_029394+NM_030984+NM_001061	0.71896431	0.252184811	0.002	0.002084	12.64418743	0.000228935	0.023363571
C3orf67	NM_198463	2.22959346	2.595403853	0.002	0.000476	12.63849774	0.00022933	0.023363571
SPG7	NM_003119+NM_199367	4.35339059	3.94476246	0.0023	0.000864	12.62400485	0.000230339	0.023363571

ZADH2	NM_175907	4.17356838	4.638105657	0.0012	0.002863	12.61509428	0.000230962	0.023363571
H3F3B	NM_005324	7.57927647	7.912376392	0.0017	0.000448	12.56068421	0.000234815	0.023624973
TTC39C	NM_153211+NM_001135993+NM_001243425	2.49072511	3.355090446	0.0011	0.01342	12.4151246	0.000245539	0.024571047
FAM81A	NM_152450	0.69148901	0.990030155	0.0009	0.000866	12.38592722	0.000247765	0.024661244
LOC100289495	NR_040022	1.04616143	1.429071413	0.0023	0.000578	12.35358247	0.000250262	0.024696913
DNAJC1	NM_022365	5.48487531	5.630294185	7E-05	0.000342	12.34695596	0.000250777	0.024696913
MGC16275	NR_026914	1.37195369	2.464931879	0.0161	0.007673	12.2792856	0.00025612	0.025090319
ARHGEF10	NM_014629	3.18630118	2.659029025	0.005	0.000515	12.25136621	0.000258367	0.025177905
PCF11	NM_015885	3.81986279	4.076721899	0.0007	0.00061	12.19128818	0.000263288	0.025523819
ZNF552	NM_024762	2.44592821	1.811413539	0.0043	0.003852	12.15854803	0.00026602	0.025590745
SKP2	NM_005983+NM_032637+NM_001243120	6.1749798	5.848884082	0.0003	0.001823	12.15013555	0.000266728	0.025590745
LOC254128+MKI67IP	NR_037858+NR_037857+NR_037856+NM_032390	5.84491249	5.206987898	0.0052	0.003092	12.12942087	0.000268481	0.025600089
AKAP8L	NM_014371	5.55256091	5.345141617	0.0008	5.83E-05	12.10827012	0.000270287	0.025600089
ZFP37	NM_003408	3.00739138	3.266313904	0.0004	0.000934	12.10052979	0.000270951	0.025600089
NDNF	NM_024574	1.71069528	2.294889522	0.0018	0.005279	12.05345709	0.000275039	0.025855014
NUMBL	NM_004756	1.93818982	1.741880848	0.0004	0.000409	12.03079273	0.000277035	0.025911777
TULP4	NM_020245+NM_001007466	2.61123048	2.903871713	0.0008	0.001006	12.00619229	0.000279222	0.025985793
PCMTD2	NM_001104925+NM_018257	4.62800114	5.094153536	0.0038	0.000752	11.93329949	0.000285834	0.026468817
PHKB	NM_000293+NM_001031835	3.3238951	2.973969784	0.002	0.000583	11.88427849	0.000290394	0.026757895
GCDH	NM_000159+NM_013976	3.47834257	3.748224145	0.0002	0.001384	11.85121729	0.000293521	0.026826579
SYT11	NM_152280	3.86959878	3.692952173	0.0004	0.000224	11.84419436	0.000294191	0.026826579
NAA16	NM_018527+NM_001110798+NM_024561	3.8688392	4.052145107	0.0007	2.95E-05	11.83091269	0.000295463	0.026826579
VPS36	NM_016075	4.24786238	3.910864915	0.0003	0.002191	11.8044369	0.00029802	0.026912245
FBXW9	NM_032301	3.15757547	2.662880929	0.0033	0.001993	11.79130585	0.000299298	0.026912245
SMAD4	NM_005359	4.22750958	3.923217378	0.0011	0.000911	11.76175695	0.000302201	0.02697201
DDX28+DUS2L	NM_018380+NM_001271762+NM_001271763+NM_017803	4.79099047	4.684836884	0.0002	7.43E-05	11.75242949	0.000303124	0.02697201
CANX	NM_001024649+NM_001746	8.44985666	8.25641848	0.0003	0.00052	11.73832571	0.000304528	0.02697201
MSL3	NM_078629+NM_078628+NM_001193270+NM_006800	3.38350526	3.596917168	0.0004	0.000612	11.7191787	0.000306447	0.02697201
CPNE8	NM_153634	3.46362963	3.79827134	0.0022	0.00026	11.70259839	0.00030812	0.02697201
ETV5	NM_004454	1.43580923	1.881132051	0.0002	0.004156	11.69247143	0.000309148	0.02697201
BCL6	NM_001706+NM_001134738+NM_001130845	2.35659334	2.032654392	0.0003	0.002051	11.68307383	0.000310106	0.02697201
DENND5B	NM_144973	2.49232126	3.016290254	0.0002	0.005954	11.61016692	0.000317668	0.027501152
QRICH2	NM_032134	0.57171537	0.441871676	0.0001	0.000265	11.58536748	0.000320293	0.027600051
ANKRD30BP2	NR_026916	0.0773227	0.043827592	1E-05	1.17E-05	11.54676344	0.000324434	0.027828103
ZWINT	NM_007057+NM_001005413+NM_032997	6.57028159	6.740631975	6E-05	0.000598	11.52938959	0.000326321	0.027860297
ATP2B3	NM_001001344+NM_021949	1.28730674	2.292968925	0.0148	0.008127	11.5158191	0.000327803	0.027860297
BOLA2+BOLA2B+LOC388242+LOC613038+SLX1A+SLX1A-SULT1A3+SLX1B+SLX1B-SULT1A4+SULT1A3+SULT1A4	NM_001031827+NM_001039182+NM_024044+NM_178044+NM_001014999+NM_001015000+NR_037608+NR_037609+NM_177552+NM_001017390+NR_002556+NR_002557	6.06852122	6.26377853	0.0001	0.000747	11.49382305	0.000330225	0.027938551
DDB2	NM_000107	5.33147323	5.653368318	0.0004	0.001981	11.47259098	0.000332584	0.028010827
FAM171B	NM_177454	3.99483417	4.190781374	5E-05	0.000837	11.41882243	0.000338655	0.028353861

ANKS1B+APAF1	NM_013229+NM_181861+NM_001160+NM_181869+NM_181868+NM_001204081+NM_020140+NM_152788+NM_001204070+NM_001204069+NM_001204068+NM_001204065+NM_001204080+NM_001204079+NM_001204067+NM_181670+NM_001204066	4.40734489	4.544616907	6E-05	0.000377	11.40965544	0.000339704	0.028353861
TMEM186	NM_015421	3.50122421	2.673830501	0.0069	0.009034	11.33902735	0.000347925	0.028750756
SERPINF1	NM_002615	3.15100134	3.549489079	0.0018	0.001951	11.33742077	0.000348115	0.028750756
EXOSC7	NM_015004+NR_023353	5.04534157	4.879298008	0.0003	0.000366	11.32916641	0.000349093	0.028750756
NSMCE4A	NM_001167865+NM_017615	5.91207238	6.258563646	0.0024	0.00046	11.29833496	0.000352777	0.028803158
RETSAT	NM_017750	4.14164257	4.53295341	0.0021	0.001456	11.29793725	0.000352824	0.028803158
SYTL4	NM_001174068+NM_001129896+NM_080737	2.30840799	1.883947279	0.0033	0.000919	11.28262326	0.000354673	0.028827595
PLA2G16	NM_001128203+NM_007069	2.78076547	3.143362187	0.0007	0.002428	11.26167916	0.00035722	0.028908413
BOLA2+BOLA2B+LOC388242+LOC613038+SLX1A+SLX1A-SULT1A3+SLX1B+SLX1B-SULT1A4+SULT1A3+SULT1A4	NM_001039182+NM_001031827+NM_178044+NM_024044+NM_001014999+NM_001015000+NR_037609+NR_037608+NM_177552+NM_001017390+NR_002556+NR_002557	6.06648012	6.262418145	0.0001	0.000815	11.13266075	0.000373433	0.030053882
LBR	NM_002296+NM_194442	6.76195074	6.24735557	0.0026	0.003856	11.12362156	0.000374604	0.030053882
BCL2	NM_000633+NM_000657	2.54252913	2.805141898	0.0015	0.000216	11.09223701	0.000378704	0.030252419
RBM14+RBM14-RBM4+RBM4	NM_001198836+NM_001198846+NM_006328+NM_001198845+NM_001198837+NM_001198843+NM_002896+NM_001198844	6.31009858	5.974709786	0.002	0.00079	11.04505839	0.000384974	0.030621904
CACNA1F	NM_001256789+NM_001256790+NM_005183	0	0.023115103	0	1.32E-05	11.01180368	0.000389473	0.030765867
KDM2A	NR_027473+NM_012308+NM_001256405	3.11136021	2.581485756	0.0003	0.006656	11.00728029	0.00039009	0.030765867
TMCO3	NM_017905	5.17903018	4.889427412	0.0011	0.000963	10.9912454	0.000392287	0.030778
CCAR1	NM_018237	4.73247019	5.48088397	0.005	0.008915	10.97271215	0.000394846	0.030778
TSPAN10	NM_031945	0.56576273	1.026217172	0.0034	0.001894	10.95122561	0.00039784	0.030778
C2orf88	NM_001042521+NM_001042520+NM_001042519+NM_032321	1.81729007	2.054734474	0.0007	0.000686	10.94959958	0.000398067	0.030778
UHMK1	NM_001184763+NM_144624+NM_175866	4.66687143	4.100896893	0.0057	0.002358	10.94642982	0.000398512	0.030778
TRIM16L	NM_001037330	4.28320325	4.513907556	0.0002	0.001181	10.92526357	0.000401495	0.03085463
PDIA3P	NR_002305	3.23774819	2.920660088	0.0019	0.000666	10.91592891	0.000402819	0.03085463
MMAB	NR_038118+NM_052845	4.24522451	3.980462788	0.0011	0.000675	10.87241864	0.000409066	0.031204711
MIR632+ZNF207	NR_030362+NM_001098507+NM_003457+NM_001032293	6.50056059	6.757618243	0.0009	0.000816	10.8224565	0.000416391	0.031568638
STK38L	NM_015000	3.50996878	2.605800328	0.0037	0.01726	10.81680894	0.000417229	0.031568638
SFXN5	NM_144579	1.84177462	2.201420279	0.0024	0.000922	10.79874719	0.000419924	0.031643939
LOC645249	NR_038835	3.82205052	3.429296631	0.0019	0.00211	10.74355416	0.000428298	0.031975022

P4HA2	NM_001142598+NM_001017974+NM_001017973+NM_004199+NM_001142599	4.59432657	4.144841397	0.0052	7.96E-05	10.73663171	0.000429363	0.031975022
GGPS1	NM_001037277+NR_036605	4.57826697	4.90813073	0.0015	0.001326	10.73592481	0.000429472	0.031975022
PAQR5	NM_017705+NM_001104554	3.54495213	3.103742236	0.0007	0.004381	10.71672187	0.000432444	0.032068073
AZIN1	NM_015878+NM_148174	6.13839548	5.619218171	0.0067	0.000468	10.63213832	0.000445851	0.032743524
STAT1	NM_007315+NM_139266	4.23113932	4.483468677	0.0007	0.001027	10.62735186	0.000446625	0.032743524
NSL1	NM_015471+NM_001042549	2.43593895	2.612412041	0.0003	0.000574	10.61603496	0.000448462	0.032743524
N4BP1	NM_153029	2.72829584	2.987516531	0.0003	0.001479	10.61525011	0.00044859	0.032743524
RAD51	NM_002875+NM_001164270+NM_133487+NM_001164269	4.3122455	4.444473329	0.0001	0.000359	10.57386834	0.000455391	0.033110138
ATP8B1+LOC100505549	NM_001242804+NM_005603	2.09573773	2.572830663	0.002	0.004182	10.50706193	0.000466647	0.033764888
GALNT2	NM_004481	4.61683062	3.973222026	0.0098	0.001443	10.49902705	0.000468025	0.033764888
ZNF33B	NM_006955	4.06369519	2.980688871	0.0137	0.018448	10.46978101	0.000473081	0.03396586
EML5	NM_183387	2.51995808	2.093524256	0.0002	0.004754	10.46187194	0.00047446	0.03396586
AFG3L2	NM_006796	5.82904945	5.561119241	0.0013	0.00068	10.44485832	0.000477444	0.034030565
IFT27	NM_006860+NM_001177701+NR_033531	3.70088163	4.284705427	0.0037	0.005672	10.43592285	0.00047902	0.034030565
INTS3	NM_023015	4.56154559	3.294325353	0.0095	0.0351	10.39545005	0.000486244	0.034412385
TSSK3	NM_052841	2.28498629	2.006722538	0.0012	0.001009	10.33368927	0.000497532	0.034912293
MARC2	NM_017898	3.56446481	4.271852845	0.0015	0.012554	10.33334076	0.000497597	0.034912293
TMEM18	NM_152834	4.35499905	4.57051293	0.0013	2.34E-05	10.32613944	0.000498935	0.034912293
ZNF814	NM_001144989	1.19858388	0.928003605	0.0021	2.51E-05	10.25131712	0.000513105	0.035708458
ZBTB25	NM_006977	3.28822307	3.128239345	3E-05	0.000704	10.24590145	0.00051415	0.035708458
SLC2A11	NM_001024938+NM_030807+NM_001024939	1.68152592	2.451565533	0.0049	0.012155	10.2249537	0.000518217	0.035771196
LOC653513	NR_037182	2.59571304	3.150120714	0.0059	0.002878	10.22147502	0.000518897	0.035771196
ZNF587B	NM_001204818	2.3106366	1.668547563	0.0027	0.009167	10.20389716	0.000522347	0.035876161
TMEM9B-AS1	NR_073431	2.13056975	1.331148202	0.0054	0.01312	10.18110028	0.000526864	0.03605339
RAD54L2	NM_015106	3.14512783	2.226339859	0.0071	0.017699	10.09710699	0.000543937	0.037085315
FBXW8	NM_012174+NM_153348	2.44953207	1.872890083	0.0023	0.007479	10.08244751	0.000546987	0.037157179
DNAJB6	NM_005494+NM_058246	5.80248008	6.105590349	0.0024	0.000349	10.07224857	0.000549122	0.037166556
NDUFV1	NM_007103+NM_001166102	6.73022251	6.374717853	0.0021	0.00169	10.05633286	0.000552474	0.037257973
LOC100288911	NR_037631	1.78702704	2.045479971	0.0005	0.00145	10.0447055	0.000554939	0.037289124
JAG1	NM_000214	3.76251278	3.40687567	0.0025	0.001281	10.03131166	0.000557796	0.037346273
FAM86B1	NM_001083537+NR_003494	3.16957794	3.471686912	0.0001	0.002608	9.979892289	0.000568937	0.037955633
NHLRC3	NM_001017370+NM_001012754+NR_073109	3.5055892	2.695030976	0.0107	0.009169	9.967172959	0.000571735	0.038006116
YPEL5	NM_001127401+NM_001127400+NM_016061+NM_001127399	4.68622618	4.938496297	0.0018	0.000158	9.944047576	0.000576868	0.038210834
PWP1	NM_007062	6.25340582	5.823749094	0.0056	2.51E-05	9.922490882	0.000581704	0.038394556
FAM156A+FAM156B	NM_001242491+NM_001099684	3.84498936	3.463846891	0.0042	0.00022	9.90302423	0.000586116	0.03844205
ZNF266	NM_001271314+NM_006631	5.12094299	4.644354381	0.005	0.00195	9.901096607	0.000586555	0.03844205
RRN3P1	NR_003370	2.88380545	3.157060494	0.0003	0.001972	9.882876851	0.000590725	0.038579537
TDRD7	NM_014290	3.32920495	3.588363794	0.0012	0.000845	9.849379049	0.00059849	0.038949988
LETM2	NM_144652+NM_001199659+NM_001199660	3.0505615	3.433008286	0.0018	0.002716	9.822143103	0.000604898	0.039100094
LINC00667	NR_015389	0.18417167	0.338165123	0.0001	0.000625	9.818544004	0.000605751	0.039100094
TFCP2L1	NM_014553	2.0627632	1.541510685	0.0022	0.006273	9.802748037	0.000609513	0.039100094
KLHL9	NM_018847	4.93852416	4.605363268	0.0013	0.00218	9.801391883	0.000609838	0.039100094
PAM	NM_001177306+NM_138821+NM_138822+NM_000919+NR_033440+NM_138766	4.78793313	4.61495198	0.0003	0.00067	9.782129311	0.000614468	0.039100094

DIP2A	NM_001146116+NM_206891+NM_015151+NM_001146115+NM_001146114+NM_206889+NM_206890	3.65563464	2.967636022	0.0003	0.014548	9.778123558	0.000615437	0.039100094
KRT15	NM_002275	0	0.062232012	0	0.000122	9.774775325	0.000616248	0.039100094
SPATA18	NM_145263	3.19663267	3.448816492	0.0018	0.000153	9.766470612	0.000618265	0.039100094
PLXNA4	NM_020911+NM_001105543+NM_181775	1.14741541	1.744763227	0.0035	0.007726	9.75563356	0.00062091	0.039100094
RHBDL3	NM_138328	3.09666002	2.902722375	0.001	0.000168	9.751983966	0.000621803	0.039100094
KIAA0368	NM_001080398	5.49197227	4.753664172	0.0088	0.008467	9.722166113	0.000629167	0.039327576
ACSM3+ERI2+LOC81691	NM_005622+NM_202000+NM_080663+NM_001142725+NM_001144924+NM_030941+NM_001199053	5.27956792	5.637208752	0.0024	0.001661	9.712904705	0.000631476	0.039327576
ATP2A1+LOC100289092+RABEP2	NM_173201+NM_004320+NR_046290+NR_046288+NR_046289+NR_046287+NM_024816	3.84289762	4.154778103	3E-05	0.003066	9.706768723	0.000633012	0.039327576
EIF3B	NM_001037283+NM_003751	6.97875121	6.852988856	0.0005	4.4E-06	9.703338994	0.000633873	0.039327576
RECQL4	NM_004260	4.47727604	4.333440911	0.0006	6.03E-05	9.627618566	0.000653248	0.040257973
GPR161	NM_153832+NM_001267611+NM_001267614+NM_001267613+NM_001267612+NM_001267610+NM_001267609	3.19243097	2.858339117	0.0019	0.001729	9.627171953	0.000653365	0.040257973
TOR3A	NM_022371	4.18115073	3.866865891	0.0015	0.001699	9.619545538	0.000655357	0.040257973
TRIM16	NM_006470	3.91097942	4.179256197	0.001	0.001295	9.602771478	0.000659767	0.040395533
KIAA1614	NM_020950	0.06810868	0.189158312	0.0004	9.78E-05	9.584921809	0.0006645	0.040551934
C2orf43	NM_021925	3.28486328	3.012381995	0.0007	0.001722	9.573580209	0.00066753	0.040603684
CXADR	NM_001207066+NM_001338+NM_001207065+NM_001207064+NM_001207063	4.14720441	4.430120452	0.0005	0.002143	9.554225827	0.000672739	0.040787283
TRIM27	NM_006510	4.80971007	5.019149684	0.0011	0.000348	9.531312872	0.000678973	0.041031552
ARHGAP11B	NM_001039841	4.28157016	4.674836776	0.0043	0.000798	9.503111083	0.000686744	0.041251681
TRPV2	NM_016113	0.40376792	0.666126554	0.0002	0.002065	9.495177944	0.00068895	0.041251681
IVNS1ABP	NM_006469	5.31659461	4.930920844	0.0037	0.001296	9.493046275	0.000689545	0.041251681
PPIL4	NM_139126	5.16794754	5.497884266	0.003	0.000608	9.480321079	0.000693106	0.041251681
DCLRE1A	NM_014881+NM_001271816	5.14423238	5.49706538	0.0018	0.002335	9.478217242	0.000693697	0.041251681
PRSS12	NM_003619	3.27331898	3.492192588	0.0003	0.001347	9.457888931	0.000699439	0.041266261
CLDN18	NM_001002026+NM_016369	0.33606689	0.485696251	0.0005	0.000295	9.45710955	0.000699661	0.041266261
CHAF1B	NM_005441	5.32812884	4.890155003	0.003	0.003422	9.434362195	0.00070616	0.041266261
MORN4	NM_001098831+NM_178832	3.0185208	3.39168344	0.003	0.001724	9.434201623	0.000706206	0.041266261
ZNF530	NM_020880	2.52926669	2.91972431	0.0031	0.002092	9.425256836	0.000708782	0.041266261
PUS7	NM_019042	4.79671861	4.957404183	0.0006	0.000267	9.42515955	0.00070881	0.041266261
ZNF417	NM_152475	2.68774717	2.886061782	0.001	0.000295	9.421487587	0.000709872	0.041266261
RNF144A	NM_014746	2.59351383	1.853402077	0.0066	0.011905	9.415251067	0.000711678	0.041266261
AKIRIN1	NM_024595+NM_001136275	5.63749829	5.74112036	0.0002	0.000212	9.395003413	0.000717584	0.041479473
MRPS5	NM_031902	5.41760512	5.228464727	0.001	0.000178	9.371221146	0.000724599	0.041650433
PTPLA	NM_014241	3.73717695	3.051050932	0.0089	0.007221	9.369812597	0.000725017	0.041650433
ZSWIM6	NM_020928	2.60136261	1.935300422	0.0022	0.013067	9.344003222	0.000732732	0.041800716
CASC1+LRMP	NM_001204127+NM_001204126+NM_006152+NM_001204102+NM_001204101+NM_001082973+NM_018272+NM_001082972	0.40258078	0.569762021	0.0005	0.000508	9.343918925	0.000732758	0.041800716
GTF2E1	NM_005513	3.13413543	3.304418728	0.0006	0.000436	9.337125142	0.000734806	0.041800716
GLOD4+RNMTL1	NM_016080+NM_018146	6.5898154	6.780696275	0.0008	0.000418	9.331139345	0.000736616	0.041800716
PAOX	NM_152911+NM_207128+NM_207127	2.05299378	2.585504417	0.0077	0.002097	9.317894163	0.000740642	0.041901416

FDPS+RUSC1+RUSC1-AS1	NM_001242825+NM_001135822+NM_002004+NM_001242824+NM_001135821+NM_001039517+NM_001105204+NM_001105203+NM_001105205+NM_014328	7.32855997	7.063406534	0.0015	0.000953	9.288484091	0.000749679	0.042241125
WWP1	NM_007013	2.69023677	2.854443786	0.0007	0.000267	9.278015434	0.000752929	0.042241125
GTPBP4	NM_012341	5.85131332	5.694685365	0.0003	0.000565	9.276328313	0.000753455	0.042241125
CSTF2T	NM_015235	3.10654692	2.981858113	0.0001	0.000399	9.26183432	0.000757988	0.042313408
KLF5	NM_001730	2.00310658	2.280356088	0.0022	0.000529	9.257687331	0.000759291	0.042313408
MIR4469+RNF170	NM_001160224+NM_001160223+NM_030954+NM_001160225+NR_027669+NR_027668+NR_039679	3.6870713	2.985337259	0.0049	0.012471	9.225367002	0.000769544	0.042670667
NOTCH2	NM_024408+NM_001200001	5.0628615	4.506920168	0.0004	0.010451	9.222734162	0.000770386	0.042670667
HPS5	NM_007216+NM_181507+NM_181508	3.26521915	2.723126408	0.0052	0.005135	9.215900651	0.000772579	0.042670667
ZNF33A	NM_006954+NM_006974	4.49150883	4.099124457	0.0028	0.002643	9.191307461	0.000780536	0.042920922
C5	NM_001735	0.47698831	0.709142205	0.0009	0.000995	9.176682501	0.000785315	0.042920922
CEP89	NM_032816	4.19572518	3.888254067	0.0013	0.002125	9.163131582	0.000789777	0.042920922
PCDHB2	NM_018936	0.41922751	0.654008816	1E-04	0.001871	9.16203919	0.000790138	0.042920922
WDR54	NM_032118	5.52376317	5.225723291	0.0017	0.001525	9.16165435	0.000790265	0.042920922
CDK4+TSPAN31	NM_005981+NM_000075	6.54470565	6.711155448	0.0005	0.00047	9.159596877	0.000790946	0.042920922
KCTD20+PXT1	NM_152990+NM_173562	4.64996538	4.351227208	0.0023	0.000895	9.151475393	0.00079364	0.042941919
GHRL+GHRLOS	NM_001134944+NR_024138+NR_024137+NR_024136+NR_024135+NR_024134+NR_024133+NR_024132+NM_016362+NM_001134941+NM_001134946+NM_001134945+NR_024146+NR_024145+NR_024144+NR_004431	1.03861048	1.678810593	0.0114	0.00338	9.132370858	0.000800022	0.043161791
BFAR	NM_016561	4.6090945	4.844322307	0.0009	0.001131	9.090311074	0.000814301	0.043802636
RASSF8	NM_001164748+NM_007211+NM_001164746+NM_001164747	4.11916135	4.353895328	0.0012	0.00082	9.083603007	0.000816607	0.043802636
AUH	NM_001698	3.23429161	4.241625931	0.0366	0.000485	9.061686353	0.0008242	0.04390173
RAE1	NM_003610+NM_001015885	6.36998013	6.531435873	0.0006	0.00032	9.053171466	0.000827174	0.04390173
ARL15	NM_019087	1.87100963	2.277012838	0.0017	0.004353	9.051145088	0.000827884	0.04390173
CLGN	NM_001130675+NM_004362	4.40970013	4.707117711	0.0006	0.002659	9.051128247	0.00082789	0.04390173
ATAD2B	NM_001242338+NM_017552	3.21893505	2.51856634	0.006	0.011946	9.043466695	0.000830579	0.043919249
FLJ37201	NR_026835	1.48148449	1.941127551	0.0045	0.003281	9.034039677	0.000833904	0.04397014
RBM23	NM_018107+NM_001077352+NM_001077351	5.07061159	4.700319239	0.0027	0.002341	9.017266458	0.000839861	0.044159131
DLEU1	NR_002605	4.50680624	4.150239678	0.0019	0.002846	8.969664333	0.000857057	0.044840059
EPM2A	NM_005670+NM_001018041	3.20768745	3.41317003	0.0006	0.000995	8.965210963	0.000858688	0.044840059
DHODH	NM_001361	2.80582597	2.968777942	0.0003	0.00065	8.955865754	0.000862124	0.044840059
SAMD13	NM_001010971+NM_001134663+NM_001134664	1.80513626	2.427619645	0.0052	0.009265	8.949595138	0.000864438	0.044840059
WHSC1	NM_001042424+NM_133331+NM_007331+NM_133335+NM_133330+NM_133334	5.62267392	5.267942495	0.0008	0.003894	8.94357835	0.000866667	0.044840059
AP1S2	NM_003916	5.26087672	5.892901891	0.0045	0.010501	8.941963786	0.000867266	0.044840059

C16orf92+FAM57B	NM_001109659+NM_001109660+NM_031478	0.75009176	1.076790041	9E-05	0.003918	8.934151988	0.000870172	0.044865696
SGOL2	NM_001160033+NM_001160046+NM_152524	4.05256803	4.182192286	0.0005	0.000119	8.882834906	0.000889569	0.044955559
SPAG9	NM_001130527+NM_001251971+NM_003971+NM_001130528	5.92799043	5.78510016	0.0001	0.000644	8.857184049	0.000899466	0.044955559
CTSZ	NM_001336	4.47010815	4.806766192	0.0018	0.002529	8.842754475	0.000905094	0.044955559
SFXN4	NM_213649	5.85134294	6.109582338	0.0018	0.000736	8.841040444	0.000905765	0.044955559
LOC728730	NR_037875	1.63223182	2.17008642	0.0092	0.001874	8.839627392	0.000906319	0.044955559
RSBN1L+RSBN1L-AS1	NR_038361+NM_198467	4.37910364	4.143963997	0.0017	0.000406	8.838873137	0.000906615	0.044955559
TTC3	NM_001001894+NM_003316	5.49793367	5.134707493	0.0032	0.001863	8.832352149	0.000909178	0.044955559
FMO4	NM_002022	0.87228067	1.240069029	0.0009	0.00433	8.823684053	0.000912599	0.044955559
WAS	NM_000377	0.18262749	0.419287884	0.0018	0.000328	8.821268647	0.000913555	0.044955559
NEMF	NM_004713	3.61818011	2.709206306	0.0023	0.029523	8.819553921	0.000914234	0.044955559
ARL16	NM_001040025	5.10480951	4.894362902	0.0001	0.001606	8.810927737	0.000917662	0.044955559
EYA4	NM_172105+NM_172103+NM_004100	3.47387481	3.63297595	0.0008	0.000133	8.803350238	0.000920687	0.044955559
CROT	NM_021151+NM_001243745+NM_001143935	2.77565231	3.136053492	0.0042	0.000846	8.797942321	0.000922853	0.044955559
RPN1	NM_002950	7.18740796	7.017003723	0.0003	0.00083	8.79675243	0.00092333	0.044955559
CEBPB	NM_005194	3.73981405	4.196854948	0.001	0.007155	8.781060919	0.000929655	0.044955559
SNAR-A10+SNAR-A11+SNAR-A14+SNAR-A3+SNAR-A4+SNAR-A5+SNAR-A6+SNAR-A7+SNAR-A8+SNAR-A9	NR_024214+NR_024215+NR_024223+NR_024224+NR_024225+NR_024226+NR_024227+NR_024228+NR_024229+NR_024242	2.59170013	3.95270842	0.0435	0.028593	8.778075672	0.000930865	0.044955559
SNAR-A10+SNAR-A11+SNAR-A14+SNAR-A3+SNAR-A4+SNAR-A5+SNAR-A6+SNAR-A7+SNAR-A8+SNAR-A9	NR_024214+NR_024215+NR_024223+NR_024224+NR_024225+NR_024226+NR_024227+NR_024228+NR_024229+NR_024242	2.59170013	3.95270842	0.0435	0.028593	8.778075672	0.000930865	0.044955559
SNAR-A10+SNAR-A11+SNAR-A14+SNAR-A3+SNAR-A4+SNAR-A5+SNAR-A6+SNAR-A7+SNAR-A8+SNAR-A9	NR_024214+NR_024215+NR_024223+NR_024224+NR_024225+NR_024226+NR_024227+NR_024228+NR_024229+NR_024242	2.59170013	3.95270842	0.0435	0.028593	8.778075672	0.000930865	0.044955559
SNAR-A10+SNAR-A11+SNAR-A14+SNAR-A3+SNAR-A4+SNAR-A5+SNAR-A6+SNAR-A7+SNAR-A8+SNAR-A9	NR_024214+NR_024215+NR_024223+NR_024224+NR_024225+NR_024226+NR_024227+NR_024228+NR_024229+NR_024242	2.59170013	3.95270842	0.0435	0.028593	8.778075672	0.000930865	0.044955559
SNAR-A10+SNAR-A11+SNAR-A14+SNAR-A3+SNAR-A4+SNAR-A5+SNAR-A6+SNAR-A7+SNAR-A8+SNAR-A9	NR_024214+NR_024215+NR_024223+NR_024224+NR_024225+NR_024226+NR_024227+NR_024228+NR_024229+NR_024242	2.59170013	3.95270842	0.0435	0.028593	8.778075672	0.000930865	0.044955559
SNAR-A10+SNAR-A11+SNAR-A14+SNAR-A3+SNAR-A4+SNAR-A5+SNAR-A6+SNAR-A7+SNAR-A8+SNAR-A9	NR_024214+NR_024215+NR_024223+NR_024224+NR_024225+NR_024226+NR_024227+NR_024228+NR_024229+NR_024242	2.59170013	3.95270842	0.0435	0.028593	8.778075672	0.000930865	0.044955559
SNAR-A10+SNAR-A11+SNAR-A14+SNAR-A3+SNAR-A4+SNAR-A5+SNAR-A6+SNAR-A7+SNAR-A8+SNAR-A9	NR_024214+NR_024215+NR_024223+NR_024224+NR_024225+NR_024226+NR_024227+NR_024228+NR_024229+NR_024242	2.59170013	3.95270842	0.0435	0.028593	8.778075672	0.000930865	0.044955559

SNAR-A10+SNAR-A11+SNAR-A14+SNAR-A3+SNAR-A4+SNAR-A5+SNAR-A6+SNAR-A7+SNAR-A8+SNAR-A9	NR_024214+NR_024215+NR_024223+NR_024224+NR_024225+NR_024226+NR_024227+NR_024228+NR_024229+NR_024242	2.59170013	3.95270842	0.0435	0.028593	8.778075672	0.000930865	0.044955559
SNAR-A10+SNAR-A11+SNAR-A14+SNAR-A3+SNAR-A4+SNAR-A5+SNAR-A6+SNAR-A7+SNAR-A8+SNAR-A9	NR_024214+NR_024215+NR_024223+NR_024224+NR_024225+NR_024226+NR_024227+NR_024228+NR_024229+NR_024242	2.59170013	3.95270842	0.0435	0.028593	8.778075672	0.000930865	0.044955559
ABCC1	NM_004996	2.84244587	1.711317922	0.0349	0.014956	8.774545791	0.000932297	0.044955559
WASF3	NM_006646	3.66725796	3.490075531	0.0012	3.67E-05	8.734118967	0.000948901	0.045637979
MEF2B+MEF2BNB+MEF2BNB-MEF2B+NR2C2AP+RFXANK	NM_001145785+NR_027308+NM_005919+NR_027307+NM_001145784+NM_001145783+NM_003721+NM_134440+NM_176880	6.21292287	6.363194759	0.0003	0.000576	8.715576219	0.00095664	0.04567589
RPP40	NM_006638	3.03318223	3.754487782	0.0163	0.004276	8.713604598	0.000957468	0.04567589
JAKMIP2+JAKMIP2-AS1	NR_038902+NM_014790+NM_001270934+NR_073101+NM_001270941	1.76918567	2.115385025	0.0009	0.003814	8.712686079	0.000957853	0.04567589
HNRNPA3+MIR4444-1	NM_194247+NR_039646	4.63974604	4.343366835	8E-06	0.003466	8.708758214	0.000959505	0.04567589
CUL3	NM_003590+NM_001257198+NM_001257197	4.29750247	3.87908058	0.0063	0.000615	8.697949781	0.000964069	0.045776085
STX12	NM_177424	4.93261954	4.395453522	0.0051	0.006432	8.681313798	0.000971147	0.045994823
LUZP6+MTPN	NM_001128619+NM_145808	6.40915028	6.204377527	0.0003	0.001364	8.670561634	0.000975756	0.046095817
ZMAT2	NM_144723	5.91936041	5.551846519	0.0002	0.005217	8.658012587	0.00098117	0.046155796
C1QTNF1	NM_153372+NM_030968+NR_049769+NM_198593+NM_198594	2.04420716	1.974836046	0.0001	5.05E-05	8.656129734	0.000981985	0.046155796
PPM1A	NM_177952+NM_021003+NM_177951	4.15231761	3.932336613	0.001	0.000974	8.637608292	0.000990052	0.046417745
KDM5B	NM_006618	4.52516177	4.132532324	0.0003	0.005959	8.620212167	0.000997704	0.046658973
OXLD1	NM_001039842	5.52100452	5.802289139	0.0023	0.000879	8.606930848	0.001003596	0.04681687
C3orf58	NM_173552+NM_001134470	4.7465117	4.322776773	0.0067	0.000535	8.599960105	0.001006705	0.046844521
LOC642361	NR_029407	2.8168197	3.179119385	0.0023	0.003001	8.591355756	0.00101056	0.046906627
TMEM194B	NM_001142645	2.22091441	1.614196072	0.0047	0.010262	8.584429173	0.001013677	0.04693424
ELMO3	NM_024712	0.67575638	1.085938281	0.0033	0.003537	8.569103858	0.001020615	0.047138212
NRP1	NM_003873+NM_001244972+NR_045259+NM_001244973+NM_001024628+NM_001024629	4.68850215	4.861426879	0.0002	0.001029	8.541721805	0.001033158	0.047393254
NRIP3	NM_020645	2.61611299	2.404610219	0.0011	0.000749	8.541611849	0.001033209	0.047393254
HIST1H2AA	NM_170745	0.30658537	0	0.0039	0	8.540385333	0.001033775	0.047393254
SCUBE3	NM_152753	0.59493836	0.386382165	0.0005	0.001342	8.518493	0.00104395	0.047722005
HGS	NM_004712	5.08782287	4.325354305	0.0011	0.022986	8.503531417	0.001050975	0.047722005
RAPGEF4	NM_007023+NM_001100397	2.55668864	3.097777083	0.0093	0.002853	8.503489315	0.001050995	0.047722005
POP1	NM_001145861+NM_001145860+NM_015029	2.64097615	2.354413923	4E-05	0.003368	8.503050014	0.001051202	0.047722005
CYP2R1	NM_024514	3.9577984	4.310022812	0.0028	0.002381	8.496704826	0.0010542	0.047741657
MAP3K13	NR_038322+NM_001242314+NM_001242317+NM_004721	4.28321766	3.940061587	0.0002	0.00467	8.485181835	0.001059671	0.047831613
GOLGA8T	NR_033933	3.24894781	3.072190948	0.0005	0.000853	8.478990739	0.001062625	0.047831613
CHTF18+GNG13	NM_022092+NM_016541	4.02351115	3.423540483	0.0098	0.005226	8.476335698	0.001063896	0.047831613
STAMBPL1	NM_020799	3.09546102	3.432171383	0.0019	0.002829	8.459460352	0.001072012	0.048080397
ACTR1B	NM_005735	5.11774426	5.29723378	0.001	0.000378	8.418587526	0.001091993	0.048858794
LOC100132707+PAXIP1	NR_024477+NR_024476+NM_007349	4.42613192	3.852823407	0.0016	0.012352	8.393091248	0.001104691	0.049066417

CCDC122+LACC1	NM_144974+NM_001128303+NM_153218	4.04171576	4.215697003	0.001	0.000323	8.390648231	0.001105917	0.049066417
LOC100129794+SLC25A21	NM_001171170+NM_030631+NR_033240	3.08713321	3.385398691	0.0037	7.2E-05	8.370212821	0.001116242	0.049066417
ATP5L	NM_006476+NR_033759	6.22841965	5.769888122	0.0034	0.005603	8.36706262	0.001117844	0.049066417
ODF2L	NM_001184766+NM_001184765+NM_020729+NM_001007022	3.64966932	4.255280417	0.0147	0.000982	8.366062669	0.001118353	0.049066417
SCCPDH	NM_016002	4.42344299	4.022680655	0.0051	0.001825	8.364227017	0.001119289	0.049066417
ZFYVE27	NM_001174122+NM_001174121+NM_001174120+NM_001174119+NM_144588+NM_001002262+NM_001002261	4.29206662	4.000688747	0.0008	0.002838	8.362862625	0.001119984	0.049066417
ITGA9	NM_002207	1.57905009	1.319178949	0.0003	0.002605	8.359262199	0.001121824	0.049066417
ACTR10	NM_018477	4.22333515	4.637490747	0.0069	0.000445	8.357601649	0.001122673	0.049066417
NANS	NM_018946	5.52989154	5.706681665	0.0011	0.000207	8.356973861	0.001122994	0.049066417
GINS2	NM_016095	5.91373078	5.27482569	0.0063	0.011278	8.344911137	0.001129191	0.049122761
DDIT4	NM_019058	7.40008872	7.533981821	0.0003	0.000493	8.344191671	0.001129562	0.049122761
KPNA2	NM_002266	7.36150942	7.520286096	1E-05	0.001075	8.334827945	0.001134404	0.049218321
PRKACB	NM_002731+NM_207578+NM_182948+NM_001242859+NM_001242858+NM_001242857+NM_001242861+NM_001242860+NM_001242862	3.43004613	3.846613627	0.0001	0.00738	8.324512576	0.001139768	0.049336029
DHCR7	NM_001163817+NM_001360	4.28212587	3.991695428	0.0029	0.000747	8.303387449	0.00115085	0.049614846
ZNF586	NM_017652+NM_001077426+NM_001204814	2.75856715	1.893686223	0.0295	0.003079	8.29790246	0.00115375	0.049614846
ZFYVE26	NM_015346	1.6425575	1.421897696	0.0009	0.001264	8.297041987	0.001154206	0.049614846
FLJ20021	NR_033874	2.09534381	2.574373198	0.0061	0.003953	8.288014816	0.001159	0.049706129

t.values: relative ratio of ON-condition to OFF-condition; q.value: adjusted p-value
